# Loss of imprinting of the *Igf2-H19* ICR1 enhances placental endocrine capacity via sex-specific alterations in signalling pathways in the mouse

**DOI:** 10.1242/dev.199811

**Published:** 2022-01-04

**Authors:** Bethany R. L. Aykroyd, Simon J. Tunster, Amanda N. Sferruzzi-Perri

**Affiliations:** Centre for Trophoblast Research, Department of Physiology, Development and Neuroscience, University of Cambridge, Cambridge CB2 3EG, UK

**Keywords:** Insulin-like growth factor, Placenta, Hormones, Endocrine cells, Trophoblast, Imprinted gene, Mouse

## Abstract

Imprinting control region (ICR1) controls the expression of the *Igf2* and *H19* genes in a parent-of-origin specific manner. Appropriate expression of the *Igf2-H19* locus is fundamental for normal fetal development, yet the importance of ICR1 in the placental production of hormones that promote maternal nutrient allocation to the fetus is unknown. To address this, we used a novel mouse model to selectively delete ICR1 in the endocrine junctional zone (Jz) of the mouse placenta (Jz-ΔICR1). The Jz-ΔICR1 mice exhibit increased *Igf2* and decreased *H19* expression specifically in the Jz. This was accompanied by an expansion of Jz endocrine cell types due to enhanced rates of proliferation and increased expression of pregnancy-specific glycoprotein 23 in the placenta of both fetal sexes. However, changes in the endocrine phenotype of the placenta were related to sexually-dimorphic alterations to the abundance of Igf2 receptors and downstream signalling pathways (Pi3k-Akt and Mapk). There was no effect of Jz-ΔICR1 on the expression of targets of the *H19*-embedded miR-675 or on fetal weight. Our results demonstrate that ICR1 controls placental endocrine capacity via sex-dependent changes in signalling.

## Abstract (precis)

**Summary:** Imprinting at *Igf2-H19* ICR1 regulates endocrine cell formation and function via sexually-dimorphic changes in Pi3k-Akt and Mapk signalling in the mouse.

## INTRODUCTION

In mammals, a subset of autosomal genes exhibit monoallelic (Ferguson-Smith and Surani, 2001) or preferential expression of one allele (Khatib, 2007; Schulz et al., 2006) in a parent-of-origin dependent manner. The expression of such imprinted genes is regulated by epigenetic mechanisms, including DNA methylation, chromatin remodelling and reciprocal expression of long non-coding RNA (Ferguson-Smith, 2011). To date, 260 imprinted genes have been identified in mice and 228 in humans, with the imprinting status of 63 genes conserved in both species (Tucci et al., 2019). Rather than being dispersed throughout the genome, imprinted genes typically colocalise in clusters, or imprinted domains, that are co-ordinately regulated by an imprinting control region (ICR) or imprinting centre (IC) (Cattanach and Kirk, 1985; Verona et al., 2003).

The first evidence of genomic imprinting came from pioneering nuclear transplantation experiments undertaken in the 1980s. The developmental failure of conceptuses carrying two paternal genomes (androgenetic) or two maternal genomes (parthenogenetic or gynogenetic) established the absolute requirement of both parental genomes for successful feto-placental development (Barton et al., 1984; Mann and Lovell-Badge, 1984; McGrath and Solter, 1984; Surani et al., 1984). Although several theories exist that attempt to explain the evolutionary origins of imprinting (reviewed by Edwards et al., 2019), the most prominent is the parental conflict hypothesis (Moore and Haig, 1991). Essential for mammalian viviparous reproduction is the substantial investment of maternal resources, including the provision of nutrients from mother to fetus throughout gestation. The parental conflict hypothesis theorises that although the father (acting through the paternal genome) is primarily interested in achieving maximal offspring growth, the mother (acting through the maternal genome) must balance supporting growth of the offspring with need for nutrients to sustain her own health, and to support future offspring. Consequently, paternally-expressed imprinted genes would be expected to promote fetal growth, whereas maternally-expressed imprinted genes would be expected to limit growth (Moore and Haig, 1991).

The placenta functions as the interface between mother and fetus during pregnancy, and it is therefore unsurprising that many imprinted genes exert their influence upon fetal growth by regulating placenta growth, development and function (Tunster et al., 2013). Indeed, placentation and genomic imprinting are thought to have co-evolved ∼168 million years ago (reviewed by Kaneko-Ishino and Ishino, 2019). Arguably the primary role of the placenta is to mediate nutrient and oxygen transfer to fetus (Burton and Fowden, 2015; Sferruzzi-Perri and Camm, 2016). However, the placenta is also a major endocrine organ, producing an abundance of hormones and signalling factors that act to adapt maternal physiology, metabolism and behaviour to support fetal growth and sustain pregnancy (Napso et al., 2018). We recently reported that the placental secretome comprises in excess of 300 proteins, including known factors like steroid hormones, prolactin/placental lactogens and pregnancy-specific glycoproteins (PSGs), as well as novel secreted placental proteins (Napso et al., 2021). Secreted placental proteins including prolactins (PRLs) and steroidogenic hormones act systemically to regulate maternal insulin production, insulin sensitivity/resistance and glucose metabolism (Ahmed-Sorour and Bailey, 1980; Brelje et al., 2004; Huang et al., 2009; Jarrett et al., 1984; Salazar-Petres and Sferruzzi-Perri, 2021; Sferruzzi-Perri et al., 2020; Wada et al., 2010). Moreover, PSGs can also act locally to promote immune-modulation and angiogenesis to support fetal development (Blois et al., 2012; Snyder et al., 2001). An imbalance in the allocation of nutrients between the mother and fetus has been linked to abnormal intrauterine development and lifelong health complications for offspring (Camm et al., 2018; Fowden et al., 2006; Gluckman et al., 2008; Sferruzzi-Perri et al., 2013a).

In humans, both transport and endocrine functions are performed by syncytiotrophoblast cells of the placenta (Dearden and Ockleford, 1983), whereas in the mouse these functions are performed by the structurally distinct labyrinth zone (Lz) and junctional zone (Jz), respectively. The Jz primarily comprises three trophoblast lineages; spongiotrophoblast (SpT), glycogen cells (GC), and trophoblast giant cells (TGC). These Jz cell types derive from a common *Tpbpa*-positive precursor (Lescisin et al., 1988; Simmons et al., 2007) and have the capacity to produce a variety of hormones, including members of the PRL family, steroidogenic hormones and PSGs (Lavoie and King, 2009; McLellan et al., 2005; Simmons et al., 2008). In addition, GC accumulate stores of glycogen and are considered to be analogous to the extravillous cytotrophoblast cells of the human placenta (Georgiades et al., 2002; Wislocki and Bennett, 1943).

Numerous mouse models exist that establish the vital role for imprinted genes in regulating placental nutrient transport (Angiolini et al., 2006; Coan et al., 2005). Perhaps key amongst these is the paternally-expressed *Igf2*, which encodes for insulin-like growth factor 2 (IGF2) and is highly expressed in placental and fetal tissues of humans (Han et al., 1987, 1988) and mice (DeChiara et al., 1991; Sferruzzi-Perri, 2018a). Consistent with the parental conflict hypothesis, paternal inheritance of an *Igf2*-null allele restricts feto-placental growth (Baker et al., 1993; DeChiara et al., 1990, 1991). This fetal growth restriction (FGR) can be attributed, at least in part, to a placental defect, with placenta-specific loss of *Igf2* also restricting feto-placental growth (Constância et al., 2002).

IGF2 is a potent promoter of cellular proliferation and differentiation, acting through the insulin receptor (INSR) or type-1 IGF receptor (IGF1R) to activate the RAS-MAPK-ERK or PI3K-AKT signalling pathways (reviewed by Czech, 1989; Forbes and Westwood, 2008; Jones and Clemmons, 1995; Sferruzzi-Perri et al., 2017; Siddle, 2011). Binding of IGF2 to INSR and activation of the PI3K-AKT signalling pathway also regulates glucose uptake and glycogen synthesis (Cross et al., 1995; Forbes and Westwood, 2008; Huang et al., 2018; Sferruzzi-Perri et al., 2016). IGF2 is thought to be cleared from the circulation via targeted lysosomal degradation following binding to the type-2 IGF receptor (IGF2R) (Czech, 1989; Lau et al., 1994; Morgan et al., 1987), which is encoded by the *Igf2r* gene that, in mice, is also imprinted, with expression derived from the maternally-inherited allele (Barlow et al., 1991).

*Igf2* localises to the ICR1 imprinted domain on chromosome 7 in mice. This domain consists of the paternally-expressed *Ins2*, *Igf2* and *Igf2as* (also known as *Igf2os*), the maternally-expressed long non-coding *H19* RNA and the microRNAs *mir-675* and *mir-483*. *Igf2* and *H19* share a common enhancer element, with imprinting mediated through paternal methylation of the differentially methylated region (DMR) ICR1 located ∼4 kb upstream of *H19* (Ferguson-Smith et al., 1993; Leighton et al., 1995b; Thorvaldsen et al., 1998; Tremblay et al., 1997). ICR1 contains recognition motifs for the zinc-finger DNA-binding protein CTCF, which blocks the interaction with enhancer elements (Bell et al., 1999; Szabó et al., 2000). Binding of CTCF to the hypomethylated maternal ICR1 prevents interaction of the *Igf2* promoter with downstream enhancers, inactivating the maternal *Igf2* allele (Bell and Felsenfeld, 2000; Kanduri et al., 2000; Szabó et al., 2000), and instead promotes the transcription of *H19* (Engel et al., 2006; Schoenherr et al., 2003). In contrast, CTCF is unable to bind to the methylated paternal allele, allowing interaction of the *Igf2* promoter with downstream enhancer elements and enabling its transcription. The absence of CTCF renders the paternal *H19* allele inactive.

Maternal inheritance of a 13 kb deletion spanning *H19* and ICR1 (*H19*^Δ13^) results in feto-placental overgrowth in mice (Leighton et al., 1995a). However, normalisation of fetal growth in mice inheriting the *H19*^Δ13^ allele maternally and an *Igf2* null allele paternally isolates this fetal overgrowth to overexpression of *Igf2* rather than loss-of-function of *H19* (Leighton et al., 1995a). Indeed, maternal inheritance of a ∼1.6 kb deletion spanning ICR1 results in re-activation of the maternal *Igf2* allele and overgrowth of neonates relative to control littermates (Thorvaldsen et al., 1998). Much of the subsequent investigation of *Igf2* function in the placenta has focused on its role in regulating nutrient transport function (Angiolini et al., 2011; Coan et al., 2008; Constância et al., 2005; Sibley et al., 2004). However, both global and placenta-specific loss of *Igf2* also restrict Jz size alongside impacting Lz size and function (Coan et al., 2008; Sferruzzi-Perri et al., 2011). Furthermore, *Igf2* is highly expressed by GC (Redline et al., 1993), with constitutive loss of *Igf2* resulting in reduced GC abundance and placental glycogen stores (Lopez et al., 1996), whereas ubiquitous maternal inheritance of the *H19*^Δ13^ allele results in an expansion of the GC population and increased placental glycogen content (Esquiliano et al., 2009).

Recent mouse studies demonstrate an emerging role for imprinted genes in regulating placental endocrine capacity (reviewed by John, 2013, 2017). For example, overexpression of the maternally-expressed imprinted genes *Phlda2* and *Ascl2* result in a reduction in Jz size (Tunster et al., 2015, 2016), suggesting that imprinting (paternal silencing) of these genes enhances placental endocrine capacity. Conversely, loss of expression of the paternally-expressed *Peg3* also restricts Jz size (Tunster et al., 2018a), suggesting that imprinting (maternal silencing) of *Peg3* would act to restrict placental endocrine capacity. We recently reported that Jz-specific loss of *Igf2* restricts placental endocrine capacity in a sexually-dimorphic manner (Aykroyd et al., 2020). We therefore hypothesised that the acquisition of imprinting of the ICR1 domain modulates placental endocrine capacity. Using a unique genetic model in which ICR1 is specifically deleted in cells of the placental Jz (Jz-ΔICR1), we sought to investigate the role of ICR1 imprinting in modulating placental endocrine function.

## RESULTS

### Validation of Jz-specific *Igf2-H19* imprinted gene dysregulation with Jz-ΔICR1

**Fig. 1.**
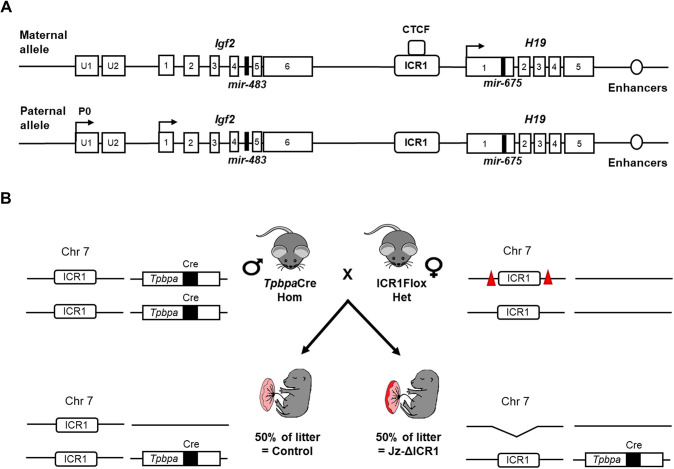
**Jz-ΔICR1 mouse genetic manipulation model.** (A) Schematic of the *Igf2/H19* gene locus. CTCF, CCCTC binding factor; ICR, imprinting control region. Arrows highlight direction of transcription of *Igf2* and *H19* alongside the location of *mir*-483 within intron 4 of *Igf2* and *mir*-675 within exon 1 of *H19*. The Lz-specific *Igf2* P0 promoter is also shown alongside the two upstream (U) exons of *Igf2*. (B) Breeding strategy to produce litters with control and Jz-ΔICR1 conceptuses. Chr, chromosome; Cre, *Cre-*recombinase; Het, heterozygous; Hom, homozygous. Triangles represent lox-P sites, arrows represent transcriptional direction.

**Fig. 2.**
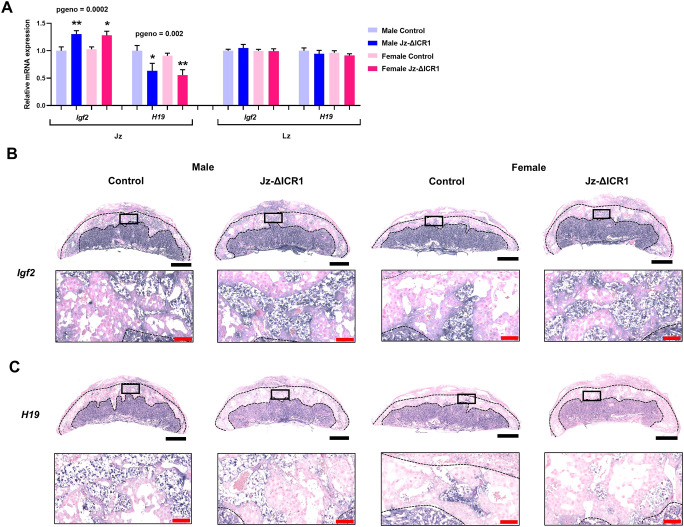
***Igf2* expression is increased and *H19* expression is decreased in the Jz of mouse placentas with Jz-ΔICR1.** (A) Expression of *Igf2* and *H19* in isolated Jz and Lz samples on D16 using qPCR (*n*=9-10 per genotype/sex in Jz and Lz, across 11 litters). Values presented as mean+s.e.m. with significance assessed by two-way ANOVA and pairwise *t*-test (*Pgenotype<0.05, **Pgenotype<0.01). (B,C) *In situ* hybridization of *Igf2* (B) and *H19* (C) in males and females. Black boxes represent the area magnified in the image below. Black bar: 1 mm. Red bar: 100 μm.

Homozygous *Tpbpa*-Cre males (Simmons et al., 2007) were mated to heterozygous ICR floxed females (LoxP sites surrounding the ICR, termed ICR1Flox; Srivastava et al., 2000) for a conditional deletion of the *Igf2* and *H19* ICR in the placental Jz (Fig. 1; Hammerle et al., 2020; Keniry et al., 2012; Waterston et al., 2002). This generated litters consisting of fetuses with control and Jz-ΔICR1 placentas (Fig. 1B). Average litter size was 7.8±0.5 (mean±s.e.m.) and control and Jz-ΔICR1 conceptuses per sex were observed around the expected Mendelian frequency (average percentage per litter: male control=28.5%, male Jz-ΔICR1=26.0%, female control=17.0% and female Jz-ΔICR1=28.5%). Using qPCR of isolated Jz obtained on gestational day (D) 16, we verified that Jz expression of *Igf2* was increased by 30% in males and 25% in females, whereas *H19* decreased by 36% in males and 39% in females (Fig. 2A). In contrast, expression of *Igf2* and *H19* was unaltered in the Lz of Jz-ΔICR1 placentas. To ensure that Jz-ΔICR1 did not result in ectopic expression of *Igf2* or *H19*, we assessed their spatial expression by *in situ* hybridisation. Consistent with previous work (Aykroyd et al., 2020; Coan et al., 2006; Redline et al., 1993) we observed high levels of *Igf2* expression in the Lz and GC, with lower levels of expression in SpT and TGC of both control and Jz-ΔICR1 placentas (Fig. 2B). In control and Jz-ΔICR1 placentas, *H19* was also widely expressed in the Lz, although expression was restricted to the GC in the Jz (Fig. 2C). Negative controls for the *in situ* hybridisations are shown in Fig. S1A,B.

### Jz-ΔICR1 affects placental structure through enhanced proliferation of endocrine cells but does not affect fetal growth

**Fig. 3.**
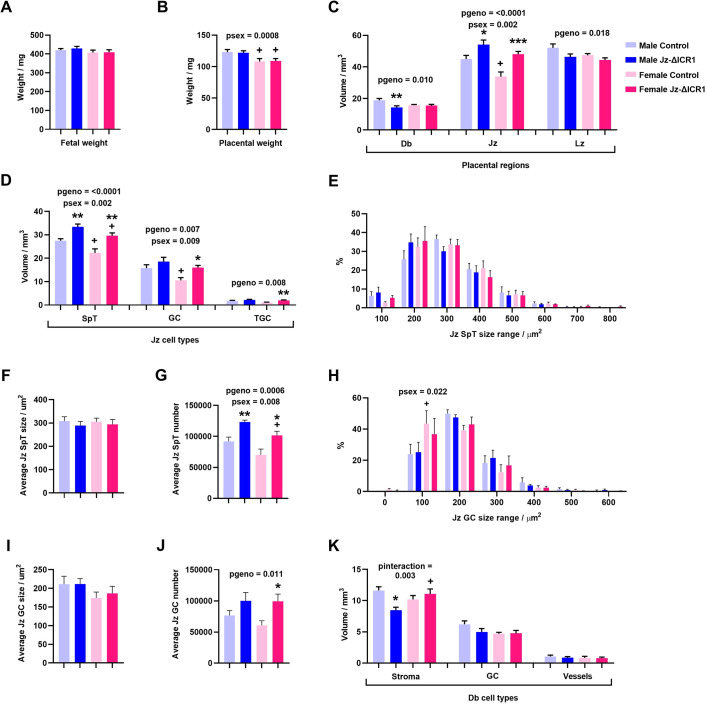
**Jz-ΔICR1 increases the formation of endocrine cells in the Jz of the mouse placenta.** (A,B) Fetal weight (A) and placental weight (B) of D16 males (control *n*=29 and Jz-ΔICR1 *n*=27) and females (control *n*=17 and Jz-ΔICR1 *n*=27) from individual pups, across 13 litters. (C,D) Volume of placental regions (C) and Jz cell types (D) (*n*=8 per genotype/sex, across seven litters). (E-J) Jz SpT cell size distribution (E), average size (F) and average number (G), and Jz GC cell size distribution (H), average size (I) and average number (J) (*n*=4 per genotype/sex, across four litters). (K) Volume of Db cell types (*n*=8 per genotype/sex, across seven litters). Values presented as mean+s.e.m. with significance assessed by two-way ANOVA and pairwise *t*-test (*Pgenotype<0.05, **Pgenotype<0.01, ***Pgenotype<0.001, +*P*sex<0.05). Db, decidua; GC, glycogen cell; Jz, junctional zone; Lz, labyrinth zone; SpT, spongiotrophoblast; TGC, trophoblast giant cell.

There was no difference in fetal weight (Fig. 3A), placental weight (Fig. 3B) or fetal to placental weight ratio (Table S1) with Jz-ΔICR1. Regardless of genotype, placentas of males were heavier but had a lower fetal to placental weight ratio when compared with females (Fig. 3B). Jz-ΔICR1 increased Jz volume by 20% in males and 43% in females. There was an overall effect of Jz-ΔICR1 to decrease Lz and decidua (Db) volume, with a significant effect of lower Db volume for male conceptuses only (Fig. 3C). Jz volume was lower in placentas of females compared with males, an effect significant in control but not in Jz-ΔICR1 conceptuses (Fig. 3C).

Further analysis of the placenta revealed that increased Jz volume with Jz-ΔICR1 was attributable to increased volume of SpT (+33%), GC (+51%) and TGCs (+96%) in females and increased volume of SpT (+22%) in males (Fig. 3D). The volume of SpT in both genotypes and volume of GC in controls was lower in female placentas compared with males. The distribution and average cell size of Jz SpT cells was unaffected by genotype (Fig. 3E,F); however, the average number of SpT cells was increased by 44% in females and 34% in males with Jz-ΔICR1 (Fig. 3G). The average number of SpT cells in Jz-ΔICR1 placentas was lower in females compared with males (Fig. 3G). The distribution and average cell size of Jz GC cells was also unaffected by genotype (Fig. 3H,I), although there was a greater percentage of GC that were within the 50-150 µm^2^ size range in control females versus control males (Fig. 3H). There was also a 63% increase in the average number of Jz GC cells in female placentas with Jz-ΔICR1, but no significant effect in males (Fig. 3J). There was an interaction between genotype and sex in determining the volume of decidual stroma (Db_S). Db_S volume was reduced by Jz-ΔICR1 in male, but not female conceptuses, and Db_S volume was greater in Jz-ΔICR1 females compared with Jz-ΔICR1 males, but not in control males compared with control females (Fig. 3K). There was no effect of genotype or sex on the volume of GC and vessels in the Db.

**Fig. 4.**
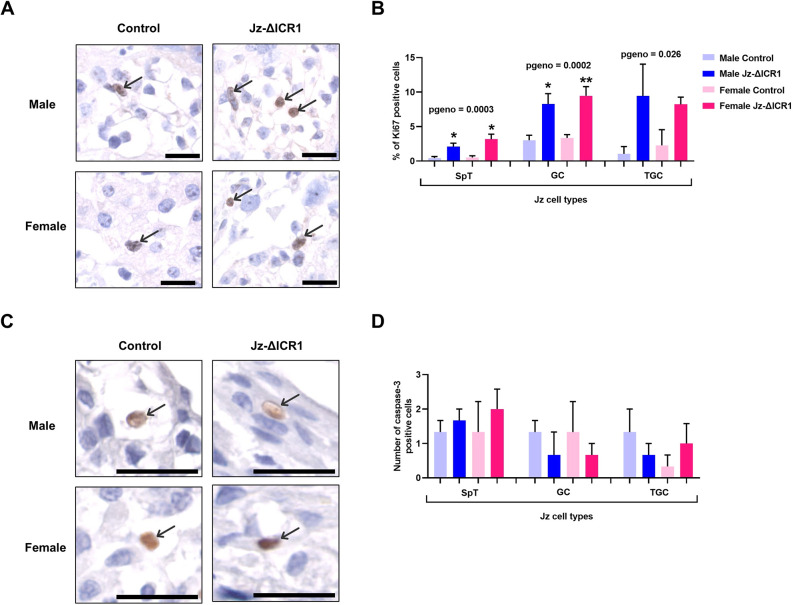
**Jz-ΔICR1 increases proliferation but does not alter apoptosis levels in the Jz of the mouse placenta.** (A-D) Immunostaining for Ki67 (A) in male and female placentas with the percentage of Ki67 positive cells (B) for each corresponding Jz cell type (positive cells divided by total number of cells for each Jz cell type) and immunostaining for cleaved caspase-3 (C) in male and female placentas with the total number of cleaved caspase-3 positive cells (D) counted for each cell type in the entire Jz section. Data were obtained on D16. Scale bar: 25 μm. Arrows indicate positive staining. Values presented as mean+s.e.m. with *n*=3-5 per genotype/sex, across five litters. Significance assessed by two-way ANOVA and pairwise *t*-test (*Pgenotype<0.05, **Pgenotype<0.01). GC, glycogen cell; SpT, spongiotrophoblast; TGC, trophoblast giant cell.

As indicated by Ki67 staining, there was an overall >3-fold increase in cell proliferation in the Jz of Jz-ΔICR1 conceptuses, an effect significant in all three Jz cell types (Fig. 4A,B). In contrast, as determined by cleaved caspase-3 staining, there was no effect of Jz-ΔICR1 on the number of cells undergoing apoptosis in the Jz (Fig. 4C,D). There was no effect of fetal sex on Jz cell proliferation or apoptosis. Representative negative control images can be found in Fig. S1C,D.

### Jz-ΔICR1 did not affect the expression of Jz cell markers but increased total placental glycogen storage in females

**Fig. 5.**
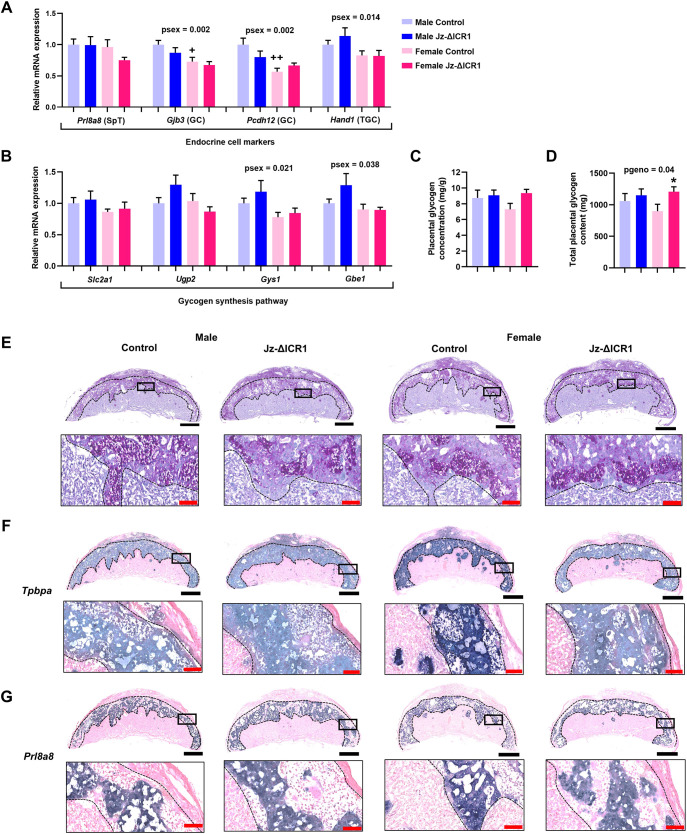
**Jz-ΔICR1 increases total placental glycogen content in females, but does not alter placental glycogen concentration or expression of glycogen synthesis pathway genes in mice.** (A,B) Jz expression of endocrine cell markers (A) and glycogen synthesis pathway genes (B) in Jz samples using qPCR (*n*=8-10 per genotype/sex, across 11 litters). (C,D) Placental glycogen concentration (mg/g; C) and total placental glycogen content (mg; D) in males and females with Jz-ΔICR1 (*n*=8 per genotype/sex, across eight litters). Data were obtained on D16. Values presented as mean+s.e.m. with significance assessed by two-way ANOVA and pairwise *t*-test (+*P*sex<0.05, ++*P*sex<0.01, *Pgenotype<0.05). (E-G) PAS stain of glycogen-containing cells (E), and *in situ* hybridization of *Tpbpa* (F) and *Prl8a8* (G) in males and females. Black boxes represent the area magnified in the image below. Black bar: 1 mm. Red bar: 100 μm.

Although volumes of the three Jz lineages were increased, expression of the SpT marker *Prl8a8*, the GC markers *Gjb3* and *Pcdh12*, and the TGC marker *Hand1* were all unaffected by Jz-ΔICR1 at the cellular level regardless of fetal sex (Fig. 5A). However, expression of *Gjb3*, *Pcdh12* and *Hand1* was lower in the Jz from females compared with males, and in the case of *Gjb3* and *Pcdh12*, pairwise comparisons revealed this was significant for controls only (Fig. 5A). We next investigated whether the increased GC volume in Jz-ΔICR1 placentas impacted placental glycogen metabolism. Jz-ΔICR1 did not affect the expression of the glucose transporter *Slc2a1* or key glycogen synthesis pathway genes in the Jz (Fig. 5B). However, overall, *Gys1* and *Gbe1* were more highly expressed in the placentas of males compared with females (Fig. 5B). Although placental glycogen concentration was not altered by Jz-ΔICR1 (Fig. 5C), total placental glycogen content was increased by Jz-ΔICR1. However, post-hoc analyses demonstrate that this is attributable to a 34% increase in the placenta of Jz-ΔICR1 females only (Fig. 5D). Visualisation of GC by PAS staining did not identify any overt differences in the spatial localisation of GC in the placenta with Jz-ΔICR1 (Fig. 5E). We further assessed the integrity of the Jz/Lz and Db/Jz boundaries by *in situ* hybridisation for the Jz marker *Tpbpa* and the SpT marker *Prl8a8* and qualitative assessment revealed no overt differences between control and Jz-ΔICR1 placentas (Fig. 5F,G).

### Jz-ΔICR1 results in increased expression of the Jz hormone *Psg23*

**Fig. 6.**
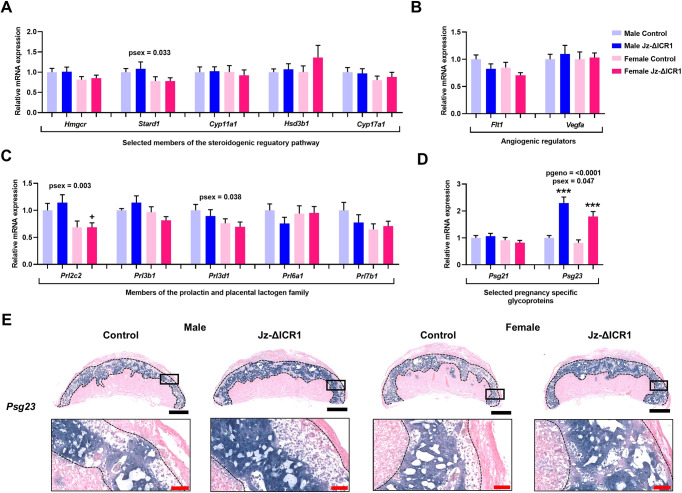
**Jz-ΔICR1 alters the expression of *Psg23*, a Jz hormone, but not steroidogenic pathway or angiogenic regulatory genes.** (A-D) Expression of steroidogenic pathway regulatory genes (A), angiogenic regulatory genes (B), and selected mouse *Prl* (C) and *Psg* (D) genes in Jz samples using qPCR (*n*=9-10 per genotype/sex, across 11 litters). Data were obtained on D16. Values presented as mean+s.e.m. with significance assessed by two-way ANOVA and pairwise *t*-test (***Pgenotype<0.001). (E) *In situ* hybridization of *Psg23* in D16 mouse placentas in males and females. Black boxes represent the area magnified in the image below. Black bar: 1 mm. Red bar: 100 μm.

We next investigated whether placental endocrine function was affected by Jz-ΔICR1. The expression of the steroidogenic pathway genes *Hmgcr*, *Stard1* (*Star*), *Cyp11a1*, *Hsd3b1* and *Cyp17a1* were unaltered by Jz-ΔICR1, although *Stard1* was expressed at a lower level in the Jz of females compared with males (Fig. 6A). Similarly, expression of members of the Prl gene family *Prl2c2*, *Prl3b1*, *Prl3d1*, *Prl6a1* and *Prl7b1* and the angiogenic regulators *Flt1* and *Vegfa* were unaffected by Jz-ΔICR1, although expression of *Prl2c2* and *Prl3d1* by the Jz was lower in females compared with males (Fig. 6B and C). Although expression of *Psg21* was unaffected by Jz-ΔICR1, we observed a >2-fold increase in expression of *Psg23* in the Jz from both males (+2.3-fold) and females (+2.2-fold) (Fig. 6D). Overall, expression of *Psg23* was lower in Jz of females compared to males (Fig. 6D). *In situ* hybridisation revealed that, in control placentas, *Psg*23 was localised to the Jz, with high levels of expression in the SpT and weak expression within GC. The spatial localisation of *Psg23* was maintained in Jz-ΔICR1 placentas, although staining intensity was notably greater when compared with controls (Fig. 6E).

### Jz-ΔICR1 alters the protein expression of Igf2 signalling factors

**Fig. 7.**
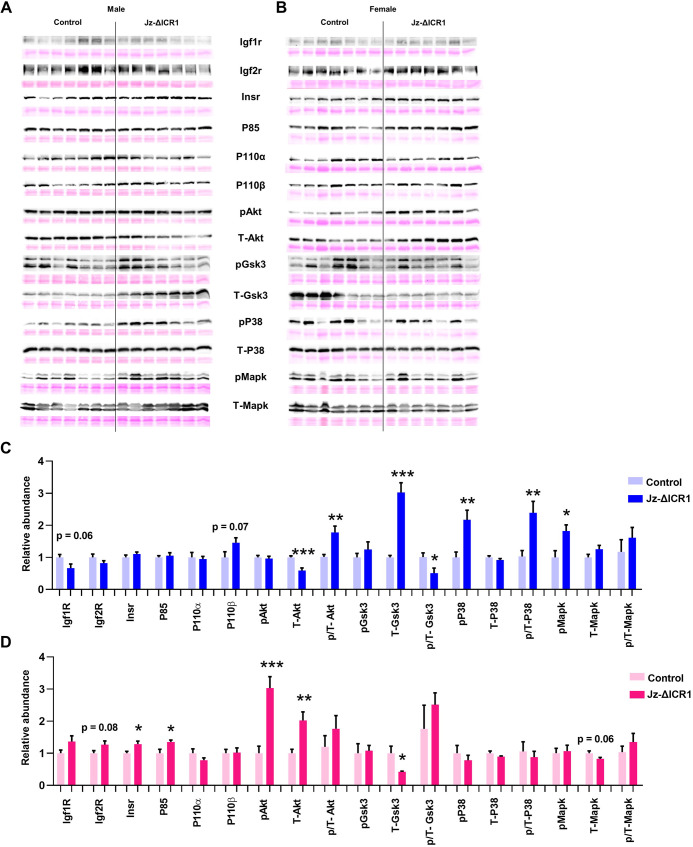
**Jz-ΔICR1 alters the protein expression of Igf2 signalling factors and downstream members of the Pi3k-Akt and Mapk pathway in the Jz.** (A-D) Representative Ponceau S staining shown to indicate protein loading with the abundance (A,B) and quantification (C,D) of Igf2 signalling proteins in males (A,C) and females (B,D). Data were obtained on D16 from *n*=7 per sex genotype/sex, across eight litters. Values presented as mean+s.e.m. with significance assessed by an unpaired two-tailed *t*-test (*P<0.05, **P<0.01, ***P<0.01).

To inform on the mechanism through which loss of imprinting of the ICR1 domain (via Jz deletion of ICR1) regulates Jz development, we quantified the abundance of Igf2 receptors (Igf1r, Igf2r, Insr) and downstream members of the Pi3k-Akt and Mapk pathways [Pi3k subunits P85, P110α, P110β, and phosphorylated (p) and total (T-) Akt, Gsk3, P38 and Mapk 42/44] using western blotting (Fig. 7). In males, p/T-Akt (phosphorylated to total Akt), T-Gsk3, pP38, p/T-P38 (phosphorylated to total P38) and pMapk were significantly increased by Jz-ΔICR1, with a tendency for an increase in P110β (*P*=0.07) (Fig. 7A,C). However, the levels of Igf1r, T-Akt and p/T-Gsk3 (phosphorylated to total Gsk3) were significantly decreased in the Jz of males with Jz-ΔICR1. Conversely, in females, there was a significant increase in Insr, P85, pAkt, T-Akt, with a trend for an increase in Igf2r (*P*=0.08) in the Jz with Jz-ΔICR1 (Fig. 7B,D). Females with Jz-ΔICR1 also had a significant decrease in the level of T-Gsk3 and a tendency for a decrease in T-Mapk (*P*=0.06), compared with controls. Changes in protein abundance with Jz-ΔICR1 were not associated with corresponding changes in gene expression as assessed by qPCR in either the Jz of males or females (Fig. S2). However, there was a significant effect of fetal sex on Jz *Insr*, *Gsk3* (*Gsk3b*), *Nras* and *Mek1* (*Map2k1*) expression (Fig. S2).

### Other contributing mechanisms

Cre-mediated ablation of the transcription factor *Tfap2c* leads to reduced Jz size, with increased expression of *H19* and decreased expression of *Psg23* in isolated Jz (Sharma et al., 2016). Other work has also shown that *Ascl2* and *Peg3* are key drivers of placental endocrine function (Tunster et al., 2016, 2018a). Thus, we examined whether the expression of *Tfap2c*, *Ascl2* and *Peg3* was altered by Jz-ΔICR1 and could contribute to the placental Jz phenotype observed. However, there was no significant effect of Jz-ΔICR1 on Jz *Tfap2c*, *Ascl2* and *Peg3* expression (Fig. S3). However, overall, the expression of *Tfap2c* and *Ascl2* by the placental Jz was ∼20% and ∼30% lower, respectively, in females compared with males (Fig. S3A,B). The decreased expression of *Ascl2* in females was also significant by pairwise comparison for control fetuses (Fig. S3B). No sex effect was seen for *Peg3* (Fig. S3C).

In addition to maternal re-activation of *Igf2* and downregulation of *H19*, Jz-ΔICR1 also results in expression of the normally-silenced maternal *mir-483* (located within intron 4 of *Igf2*) and reduced expression of *mir-675* (located within exon 1 of *H19*) (Hammerle et al., 2020; Keniry et al., 2012; Waterston et al., 2002). Although no miR-483 targets have been identified, some for miR-675 have been reported, including *Igf1r*, *Rap1gap*, *Egr3* and *Slc44a1* (Keniry et al., 2012). However, the expression of these by the Jz was not affected by Jz-ΔICR1 (Fig. S2A and Fig. S4). Although *Rap1gap* was expressed at a lower level in females compared with males, an effect significant by pairwise comparison for controls (Fig. S4).

### Jz-ΔICR1 exerts an indirect effect on the placental Lz

**Fig. 8.**
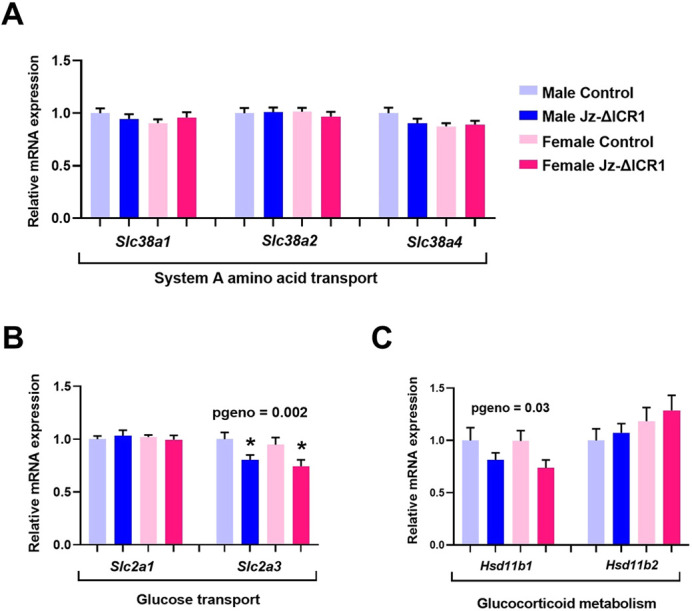
**Jz-ΔICR1 reduces glucose transporter and glucocorticoid enzyme gene expression in the placental Lz.** (A-C) Expression of system A amino acid transporters (A), glucose transporters (B) and glucocorticoid enzymes (C) in Lz samples with Jz-ΔICR1 using qPCR (*n*=9-10 per genotype/sex, across 11 litters). Data were obtained on D16. Values presented as mean+s.e.m. with significance assessed by two-way ANOVA and pairwise *t*-test (*Pgenotype<0.05).

As Jz-ΔICR1 led to a reduction in Lz volume, this suggested there could be some indirect effects of changes in Jz phenotype (e.g. glycogen content and hormone expression) on Lz functional capacity. To explore this, we assessed the expression of key glucose (*Slc2a1*, *Slc2a3*) and amino acid (system A; *Slc38a1*, *Slc38a2*, *Slc38a4*), transporters in separated Lz by qPCR (Fig. 8). As the Lz also performs a role in regulating the fetal exposure of maternal glucocorticoids we also quantified the gene expression of key glucocorticoid metabolising enzymes (*Hsd11b1*, *Hsd11b2*) (Fig. 8). Although there was no significant change in the expression of the system A amino acid transporters with Jz-ΔICR1 (Fig. 8A), glucose transporter *Slc2a3* and glucocorticoid activating enzyme *Hsd11b1* expression were reduced regardless of fetal sex when compared with controls (Fig. 8B,C). The expression of *Cd9*, the only known Psg receptor (Waterhouse et al., 2002), was also measured in the Lz, to explore whether it may be altered and may provide some explanation for the indirect effects of Jz-ΔICR1 on the Lz. However qPCR analysis revealed its expression by the Lz was not significantly altered in either sex by Jz-ΔICR1 (Fig. S5).

## DISCUSSION

Emerging studies demonstrate a role for imprinted genes in regulating placental endocrine capacity (John, 2013, 2017), with constitutive gene manipulations showing that the maternally-expressed genes *Ascl2* and *Phlda2* restrict Jz size (Tunster et al., 2015, 2016), and paternally-expressed genes *Peg3* and *Igf2* appear to enhance Jz size (Esquiliano et al., 2009; Tunster et al., 2018a). Furthermore, we recently reported that conditional loss of *Igf2* in cells of the Jz affects placental endocrine capacity in a sexually-dimorphic manner (Aykroyd et al., 2020). In the present study, we generated a novel loss-of-imprinting (LOI) model to investigate the role of imprinting of the ICR1 domain in modulating placental endocrine capacity. We used *Tpbpa*-Cre-mediated deletion of ICR1 to drive LOI of the ICR1 domain specifically in cells of the Jz of the mouse placenta (Jz-ΔICR1). Quantification of gene expression in isolated Jz samples revealed a ∼30% increase in *Igf2* expression and a concomitant ∼40% reduction in *H19* expression within the Jz following maternal transmission of the floxed allele. Although the magnitude of changes in *Igf2* and *H19* expression may partly reflect the presence of *Tpbpa*-negative cells in isolated Jz samples, a similar level of *Igf2* re-activation and *H19* suppression has been seen in another study involving a 1.6 kb deletion spanning ICR1 (Thorvaldsen et al., 1998). Moreover, our findings reinforce the idea that there may be additional mechanisms controlling the placental expression of these imprinted genes from the maternal allele (Kaffer et al., 2001; Nordin et al., 2014; Sasaki et al., 2000). Importantly, correct spatial localisation of *Igf2* and *H19* transcripts was maintained in Jz-ΔICR1 placentas, and there was no effect on Lz expression of *Igf2* or *H19*. However, we cannot rule out an effect of potential changes in allele-specific DNA methylation and histone modifications with Jz-ΔICR1, which could be explored in future work.

Although placental weight was unaffected, Jz-ΔICR1 resulted in a slight reduction in Lz and Db volumes with a concomitant increase in Jz volume in placentas of both males and females. When analysed independent of sex, the increased Jz volume was attributable to increased volumes of all three Jz constituent cell types (SpT, GC and TGC), with no effect on average size of SpT or GC. This is consistent with findings in the *H19*^Δ13^ model, in which cell size was unaltered (Esquiliano et al., 2009), and suggests that the increased volume of Jz cell types is related to an expansion in cell number. As expression of *Igf2/H19* was largely restricted to the GCs in the Jz, yet the volume of TGC and SpT was also affected by Jz-ΔICR1, these data suggest a role of paracrine IGF2 from the GC in driving the expansion of other cell types in the Jz. Indeed Igf2 receptors are localised throughout the Jz cell types of the mouse placenta, which would be permissive for paracrine signalling (Charnock et al., 2016). Although Igf2 may have an anti-apoptotic function in the placenta (Sferruzzi-Perri et al., 2017), the level of the apoptotic marker caspase-3 was unaltered in Jz cell types by Jz-ΔICR1. However, there was more than a 3-fold increase in the percentage of SpT, GC and TGC positive for the proliferation marker Ki67 in Jz-ΔICR1 placentas compared with controls. In addition to the well-established role of IGF2 in promoting trophoblast proliferation (Chen et al., 2016; Forbes et al., 2008), *H19* has also been linked to regulating placental cell proliferation. A Jz-specific manipulation that results in upregulated *H19* expression also led to a 75% reduction in the number of Ki67-positive cells compared with control placentas at D12.5 (Sharma et al., 2016). Taken together, these data show that the enhancement of Jz growth observed with Jz-ΔICR1 is due to an increase in proliferation and not a change in Jz cell size or apoptosis, which occurs as a result of both *Igf2* and *H19* mis-expression.

In addition to mediating the transfer of nutrients from mother to fetus, the placenta also stores glucose as glycogen (Lefebvre, 2012; Tunster et al., 2020). These placental glycogen stores are thought to provide an important source of energy to support fetal growth during late gestation (Coan et al., 2006). Although Jz-ΔICR1 did not affect expression of key glycogenesis pathway genes measured, or weight-adjusted glycogen concentration within the Jz, total placental glycogen content was increased. This suggests that the increased glycogen content simply reflects the increased Jz size. These findings are consistent with previous work showing that manipulation of the *Igf2-H19* locus affects placental glycogen levels (Esquiliano et al., 2009; Lopez et al., 1996). The effect of Jz-ΔICR1 to increase total placental glycogen was most pronounced for females and, overall, reflected greater GC number. These data indicate that imprinting of ICR1 in the Jz acts, at least in part, to restrict placental glycogen storage, thus reducing the necessity of the mother to invest resources that she may otherwise require for maintenance of health. As GC accumulate glycogen from D12 (Bouillot et al., 2006), it would be interesting to assess placental glycogen content at an earlier time point, for example D13, to determine when the phenotype develops. In addition, glycogen cells originate in the ectoplacental cone, before undergoing rapid proliferation and migrating into the Db from D14.5 (Coan et al., 2006). Although we observed an increased volume of glycogen cells in the Jz, the total volume of glycogen cells in the Db was unaltered. Future work should explore whether the mechanisms regulating GC invasion into the Db may be altered by Jz-ΔICR1.

Despite the morphological changes, expression of markers of Jz cell lineages and various endocrine-related genes were largely unaltered at the cellular level by Jz-ΔICR1. However, as the abundance of the cell types expressing these genes is increased, total placental expression/output of such endocrine mediators would be predicted to be increased. For example, although expression of *Prl8a8* appears to be unchanged, this simply reflects that the level of *Prl8a8* expression per cell is unaltered and gives no indication as to the abundance of *Prl8a8*-expressing cells, which was assessed instead by stereology. A notable exception to this was *Psg23* expression, which increased >2-fold in the Jz of both males and females with Jz-ΔICR1. This indicates that the level of *Psg23* expression by *Psg23*-expressing cells is increased via a molecular mechanism, rather than a change in cell abundance. However, the mechanism underlying *Psg23* upregulation is unclear. PSGs are thought to have a predominantly immune-modulatory function, and Psg23 is one of the most abundant Psgs expressed in the mouse placenta in late gestation (McLellan et al., 2005). Human PSGs induce the production of anti-inflammatory cytokines *in vitro* (Snyder et al., 2001). In addition, murine Psg23 and human PSG1 share a common function in promoting feto-placental blood supply via induction of vascular remodelling and angiogenesis (Lisboa et al., 2011; Wu et al., 2008). Although, the expression of the angiogenesis regulators *Flt1* and *Vegfa* were unaffected by Jz-ΔICR1, given the notable upregulation of *Psg23* expression, future work should evaluate whether maternal spiral artery remodelling, inflammatory cytokine production, placental Lz morphology and utero-placental blood flow may be altered by Jz-ΔICR1. Further work is also required to understand the mechanisms by which some hormone genes are altered by Jz-ΔICR1 and others are not. Indeed, manipulations of other genes, like the Pi3k pathway, through which Igf2 can signal, also exerts changes in select placental hormone genes (Sferruzzi-Perri et al., 2016). There are also examples in the literature showing that manipulation of other imprinted genes, like *Phda2* (Tunster et al., 2015), *Peg3* (Tunster et al., 2018a), *Ascl2* (Tunster et al., 2018b) and *Cdk1nc* (Tunster et al., 2011) have specific effects on placental hormone gene expression. Taken together, Jz-ΔICR1 appears to increase placental endocrine capacity and function through two mechanisms; by increasing the abundance of the endocrine cell lineages (SpT, GC and TGC), and by driving increased expression of *Psg23* at the cellular level.

The mechanism underlying enhanced *Psg23* with Jz-ΔICR1 is unclear. Interestingly, *Tpbpa*-Cre-mediated ablation of the transcription factor *Tfap2c* resulted in a reduction in Jz size, with increased *H19* and decreased *Psg23* expression in the Jz (Sharma et al., 2016). However, expression of *Tfap2c* was unaltered in response to Jz-ΔICR1, which argues against a role for altered *Tfap2c* in our model. There was also no change in the expression of *Ascl2* or *Peg3*, key drivers of placental endocrine function, with Jz-ΔICR1. Maternal re-activation of *Igf2* and downregulation of *H19*, will also result in expression of the normally-silenced maternal *mir-483* (located within intron 2 of *Igf2*), and reduced expression of *mir-675* (located within exon 1 of *H19*). miRNAs can regulate gene expression and have been implicated in Jz cell proliferation and differentiation (Sharma et al., 2019). As miR-675 has been linked with placental growth suppression (Keniry et al., 2012), dysregulation of miR-483 or miR-675 could contribute to the phenotypes observed in response to Jz-ΔICR1. However, we did not observe any alterations in expression of several miR-675 targets, including *Igfr1*, in response to Jz-ΔICR1. Furthermore, expression of *Psg23* is unaltered in the *H19*^Δ13^ model (Keniry et al., 2012), suggesting that *Psg23* is not a target of miR-675. No miR-483 targets have been identified to date, thus the contribution of perturbed miR-483 expression to the phenotype observed with Jz-ΔICR1 requires study.

We wanted to further explore the underlying molecular mechanisms through which imprinting of ICR1 may exert a regulatory influence on placental endocrine capacity. Since *Igf2* is elevated ∼30% in the Jz due to Jz-ΔICR1, and the phenotype in the *H19*^Δ13^ model was attributed to elevated Igf2 (Leighton et al., 1995a), we focused our attention on the abundance of receptors that bind to, and signalling pathways downstream of, Igf2. In females, protein levels of Insr, Pi3k-P85, phosphorylated Akt and total Akt were increased alongside decreased levels of total Gsk3 in the placental Jz response to Jz-ΔICR1. As the Pi3k-Akt signalling pathway inhibits Gsk3, which is a negative regulator of glycogen synthesis (Cross et al., 1995; Diehl et al., 1998), our findings are consistent with increased placental glycogen synthesis in Jz-ΔICR1 females. Deletion of a negative regulator of Pi3k-Akt signalling has been shown to increase placental Jz size in mice (Church et al., 2012). This signalling pathway is also implicated in the proliferation and differentiation of individual trophoblast cells in the placenta, notably GC and TGC (Kent et al., 2010; Lee et al., 2019; Sferruzzi-Perri et al., 2016). Thus, enhanced Pi3k-Akt activation (via Insr) could also explain the increase in Jz formation in females with Jz-ΔICR1.

In males with Jz-ΔICR1, there was no increase in the abundance of signalling receptors for Igf2 (there was even a trend for reduced Igf1r), and abundance of total Akt was decreased and levels of total Gsk3 increased in the placental Jz. However, there was a trend for elevated Pi3k-P110β, and the ratio of active phosphorylated to total Akt was increased, whilst the ratio of inactive phosphorylated to total Gsk3 increased in Jz-ΔICR1 males. These changes all suggest enhanced Pi3k-Akt signalling. However, unlike in females, this enhanced Pi3k-Akt signalling was not associated with an increase in total placental glycogen or GC and TGC abundance in Jz-ΔICR1 males. These data collectively suggest that the precise mechanism through which activation of Pi3k-Akt signalling occurs, or the existence of other signalling pathways, are important for modulating glycogen levels and Jz morphogenesis in the female and male placenta. Indeed, males, but not females, showed an increased level of phosphorylated Mapk, phosphorylated P38 and phosphorylated to total P38 ratio in response to Jz-ΔICR1. Members of the MAPK pathway are activated by IGF2 to promote cell proliferation (Forbes and Westwood, 2008). P38 signalling also regulates programmed cell death pathways (Cheng and Feldman, 1998; Li et al., 2003) and is important for murine placental Jz formation, particularly SpT differentiation (Mudgett et al., 2000). While there was no change in apoptosis with Jz-ΔICR1, elevated abundance and activation of the Mapk pathway could also explain the enhanced Jz formation in males in response to Jz-ΔICR1. Changes in Jz protein abundance with Jz-ΔICR1 were not linked to alterations in gene expression, highlighting a role for post-transcriptional regulation in mediating the changes seen for males and female fetuses, which require study in further work.

Previous studies manipulating the expression of genes within the ICR1 domain have demonstrated profound effects on fetal growth. For instance, maternal inheritance of the *H19*^Δ13^ allele, which deletes both *H19* and ICR1 and results in maternal reactivation of *Igf2*, causes a 27% increase in fetal weight (Leighton et al., 1995a). Similarly, maternal inheritance of a deletion spanning only the ICR1 results in a 17% increase in neonatal weight (Thorvaldsen et al., 1998), whereas targeted deletion of *H19* (*H19*^Δ13^), which results in partial (∼25%) re-activation of maternal *Igf2*, enhanced fetal weight by ∼8% (Ripoche et al., 1997). Despite a ∼30% increase in *Igf2* expression in the Jz and the enhanced placenta endocrine and glycogen storage capacity, fetal weight was not increased in our Jz-ΔICR1 model. There are several potential explanations for this observation. Firstly, deletion of ICR1 was specifically targeted to cells of the Jz, and *Igf2* expression in the Lz or fetus (which are unaltered) may be relatively more important for fetal growth. This notion is in line with previous studies using manipulations that only affect one compartment of the placenta or fetus (Aykroyd et al., 2020; Sandovici et al., 2019 preprint; Sferruzzi-Perri et al., 2011). Secondly, we assessed impacts on fetal weight on D16 of pregnancy only. This day was chosen as it is when the placental Jz is at its largest size (Coan et al., 2006) and mouse dams are most insulin resistant to favour fetal nutrient supply (Musial et al., 2016). Hence, work is required to explore whether changes in placental endocrine phenotype with Jz-ΔICR1 offer benefit when the fetus enters its exponential growth phase in the lead up to term. It will also be interesting to explore whether the increased glycogen content of Jz-ΔICR1 placentas may have implications for placental use of glucose for metabolic processes and/or fetal glucose supply in late gestation. This could be investigated in future work by performing placental metabolic and transport assays and quantifying fetal glucose concentrations (Sferruzzi-Perri, 2018b; Sferruzzi-Perri et al., 2019). Thirdly, we generated litters of mixed genotypes, such that both control and Jz-ICR1Δ conceptuses were exposed to the same *in utero* environment. Although this approach serves to normalise variations in the maternal environment, control littermates will be exposed to the potentially altered *in utero* environment caused by enhanced endocrine capacity of Jz-ICR1Δ placentas and may not show the normal pattern of fetal growth. Indeed, the phenotype of genetically wild-type littermates has been shown to be influenced by mutant littermates with phenotypes affecting placental endocrine function (López-Tello et al., 2019; Tunster et al., 2016). Future work may address any possible dilution of an effect on fetal growth by undertaking comparisons between litters comprised entirely of Jz-ΔICR1 or wild-type conceptuses.

The manipulation model used in this study was Jz-specific, so it was particularly interesting to see that there was an indirect effect of Jz-ΔICR1 on the Lz (Lz volume and expression of *Slc2a3* and *Hsd11b1* genes were reduced compared with controls). This is consistent with other work suggesting that the Jz produces signals that regulate the Lz (Tanaka et al., 1997). *Slc2a3* expression is localised specifically to the Lz in the rodent placenta (Zhou and Bondy, 1993) and is involved in transporting glucose to the fetus for growth and development (Ganguly et al., 2007). The *Hsd11b1* gene encodes an enzyme that converts inactive cortisone/11-dehydrocorticosterone to active cortisol/corticosterone (Tomlinson et al., 2004), facilitating fetal exposure to maternal glucocorticoids that can slow fetal growth (Vaughan et al., 2012). As decreased Lz *Slc2a3* would be expected to reduce fetal growth, whereas downregulated Lz *Hsd11b1* would be predicted to enhance fetal growth, the combination of both of these changes with Jz-ΔICR1 may be neutralised and explain why fetal weight is not altered compared with controls. It is interesting to note that the Lz expression of *Slc2a3* and *Hsd11b1* were not altered with global deletion of *H19* alone (*H19*^Δ13^*)* (Keniry et al., 2012), whereas a 20% decrease in the expression of *Slc2a3* was observed with global deletion of both *H19* and ICR1 (H19^Δ13^) in accompaniment with increased *Igf2* (Angiolini et al., 2011). This indicates that *Igf2* is involved in regulating Lz nutrient transport capacity independently from *H19*. Moreover, the reduction in Lz size and glucose transport capacity may reflect an attempt to compensate for the enhanced endocrine capacity of Jz-ΔICR1 placentas with the aim of ensuring normal conceptus growth. Further work is required to understand the method by which the Lz may be affected by Jz-ΔICR1 and the resultant impacts on placental endocrine capacity.

In light of accumulating evidence of sexual dimorphism in placental adaptations to genetic and/or environmental perturbations (Aykroyd et al., 2020; Barke et al., 2019; Kalisch-Smith et al., 2017b; Napso et al., 2019; Rosenfeld, 2015) and offspring outcomes (Christoforou and Sferruzzi-Perri, 2020; Dearden et al., 2018; Rodgers and Sferruzzi-Perri, 2021), we accounted for fetal sex in our analyses. Whereas Jz size was increased in placentas of both male and female conceptuses in response to Jz-ΔICR1, the underlying mechanisms appear to exhibit a degree of sexual dimorphism. For example, the increase in Jz volume of Jz-ΔICR1 females was attributed to increased volumes of all three Jz cell types, whereas statistical significance was achieved only for SpT in Jz-ΔICR1 males. Furthermore, abundance of individual components of the Pi3k-Akt and Mapk signalling pathways in the Jz with Jz-ΔICR1 was dependent on fetal sex. We also found that placental weight and Jz size were greater in males compared with females. This is consistent with previous findings in rodents and humans (Eriksson et al., 2010; Kalisch-Smith et al., 2017a). In our study, males also exhibited increased expression of several Jz lineage markers (*Gjb3*, *Pcdh12*, *Hand1*), glycogen synthesis genes (*Gys1*, *Gbe1*, *Gsk3*), growth regulators (*Insr*, *Nras*, *Mek1*, *Tfap2c*, *Rap1gap*) and endocrine-related genes (*Stard1*, *Prl2c2*, *Prl3d1*, *Psg23*) compared with females, irrespective of genotype. In addition, we noted that males exhibited lower fetal:placental weight ratios compared with females. Further work is required to elucidate the causal mechanisms behind the sex-dependent differences in the placenta. Sex hormones may be likely candidates, with variation in sex hormone production by the fetal gonads and adrenal glands in mice between females and males (Kalisch-Smith et al., 2017b). Genetic factors may also contribute. Coding genes on the Y chromosome (Gubbay et al., 1990; Yamauchi et al., 2014) and X inactivation escape genes like *Slc38a5* (an amino acid transporter; Finn et al., 2014), may influence placental functional capacity in the two fetal sexes. Moreover, recent published work in the early human placenta showed an enrichment of pathways essential for protein synthesis, cell growth and energy metabolism in males compared with females which were largely linked to genes encoded by the X or Y chromosome (Gonzalez et al., 2018). Taken together, our findings further emphasise the need to control or account for fetal sex in studies of fetal and placental developmental physiology.

In summary, Jz-ΔICR1 enhances endocrine cell formation and Jz hormone expression. Although this phenotype is observed in both sexes, the signalling pathway response mechanisms attributable to these alterations are sexually dimorphic. Moreover, the expansion of the Jz occurs at the expense of Lz size and is further accompanied with changes in Lz functional genes. However, despite changes in placental phenotype, fetal weight was not affected by Jz-ΔICR1. The influence of altered placental phenotype with Jz-ΔICR1 on fetal and maternal physiology is yet to be determined. In addition, the effects of Jz-ΔICR1 in later gestation (for example D19), when fetal growth demands are highest is yet to be explored. Studies of the human placenta have reported perturbations in the regulation of the *Igf2-H19* locus and alterations in the expression/abundance of Igf2 signalling factors and placental hormones in pregnancy complications including gestational diabetes, fetal growth restriction and large for gestational age (Laviola et al., 2005; Le et al., 2013; Liao et al., 2017; Ngala et al., 2017; Su et al., 2016). Therefore, this study may provide valuable insight for understanding the pathogenesis of human pregnancy conditions. In addition, the magnitude of changes in *Igf2* and *H19* expression seen in this study supports the notion that there may be other regulatory mechanisms controlling the expression of these genes from the maternal allele in the placenta. Finally, our study highlights that maternal and paternal imprinted genes may govern the allocation of resources to the fetus additionally via modulation of placental endocrine function in pregnancy.

## MATERIALS AND METHODS

### Maintenance of transgenic mice

The experiments for this study were approved by the University of Cambridge Animal Welfare and Ethical Review Body performed under the UK Home Office Animals (Scientific Procedures) Act 1986. Homozygous *Tpbpa*-Cre males (Simmons et al., 2007) were mated to heterozygous ICR floxed females (LoxP sites surrounding the ICR, termed ICR1Flox; Srivastava et al., 2000) to generate litters containing fetuses with control and Jz-ΔICR1 placentas (Fig. 1). The transgenic mice were maintained on a C57BL/6NCrl (Charles River, UK) background for >10 generations. Mice were housed under a 12 h light/12 h dark photocycle, 22°C air temperature and 21% oxygen saturation with access to water *ad libitum* and standard laboratory chow (Rat and Mouse No.3; Special Diets Services).

### Tissue collection

Dams were killed by cervical dislocation on D16 of pregnancy (presence of a copulatory plug denoted D1 and term occurs ∼D20). Placentas and fetuses were dissected from the dam uterus and weighed. Placentas were bisected on the short axis, one half was separated into individual Jz and Lz, as described previously by Sferruzzi-Perri et al. (2009), snap-frozen in liquid nitrogen and stored at −80°C for either gene expression or western blotting analysis. The remaining placental half was kept whole and either snap frozen in liquid nitrogen and stored at −80°C for placental glycogen content analysis or fixed in 4% paraformaldehyde, dehydrated, embedded into paraffin wax and sectioned exhaustively from the mid-line at 8 µm for histological analysis. Fetal tails were collected for PCR to establish the Flox genotype (FPrimer: 5′-CAGGCCTGTCCTCACCTGAAC-3′, RPrimer: 5′-GCCAGCTTGCCTTGGCAACCCCTT-3′) and sex with *Sry* genotyping (FPrimer: 5′-GTGGGTTCCTGTCCCACTGC-3′, RPrimer: 5′-GGCCATGTCAAGCGCCCCAT-3′ with a PCR autosomal gene control FPrimer: 5′-TGGTTGGCATTTTATCCCTAGAAC-3′, RPrimer: 5′-GCAACATGGCAACTGGAAACA-3′). A single placenta from each of the four possible sex and genotype combinations (male control; male Jz-ΔICR1; female control; female Jz-ΔICR1) from each litter was selected for further analysis, where possible. Where multiple placentas of the same sex and genotype combination were present in the same litter, the placenta with the weight closest to the litter mean for that combination was selected.

### Stereological analysis

Every 20th paraffin-embedded placental section was stained with haematoxylin and eosin (*n*=8 per genotype/sex, across eight litters). Images of each placental section were captured at 40× magnification using a NanoZoomer 2.0-RS (Hamamatsu). The gross structure of each placental zone (Db, Jz and Lz) and the proportion of cells in the Jz and Db were analysed using the newCAST System (Visiopharm) as described by Aykroyd et al. (2020). The average size of Jz GC was estimated by using the freehand annotation tool in the NDP.view2 (Hamamatsu) and measuring the area of 100 Jz GC and SpT in a mid-line section (*n*=4 per genotype/sex, across four litters). A histogram was produced for cell size distribution of SpT and GC with bin widths of 100 µm^2^. The average number of SpT and GC in the Jz was determined by dividing the SpT and GC volume by average cell size. A qualitative assessment of Jz interdigitation into the Lz, and Jz boundary integrity with the Db and Lz, was also performed (*n*=4 per genotype/sex, across four litters).

### *In situ* hybridisation

The expression of *Igf2*, *H19*, *Tpbpa*, *Prl8a8* and *Psg23* was localised in placental sections using *in situ* hybridisation. Previous studies have described the generation of probes for *Igf2* and *Prl8a8* (Aykroyd et al., 2020) and *Tpbpa* (Lescisin et al., 1988). A 659 bp region of *Psg23* was amplified by PCR from wild-type placental cDNA (primer sequences: 5′-GCTGTGACCCTCTTGACTCT-3′, 5′-AAATGCCTCTGCCCTGCTAT-3′), cloned into the pDrive vector system (Qiagen), with a linearised vector used as a template for probe transcription. For *H19*, the template for probe transcription was generated by PCR amplification of a 405 bp fragment from wild-type placental cDNA using primers incorporating a T3 (FPrimer: 5′-AATTAACCCTCACTAAAGGGTTGTCGTAGAAGCCGTCTGT-3′) or T7 (RPrimer: 5′-TAATACGACTCACTATAGGGGACAGGAGGGAGATGATGAAGT-3′) RNA Polymerase binding site, as described by Langford et al. (2018). The amplicon was purified using the Monarch PCR and DNA Cleanup Kit (New England Biolabs), and 1 μg used as template for transcription of digoxigenin-labelled riboprobes using the DIG RNA Labelling Mix (Sigma-Aldrich).

Probe hybridisation was performed overnight at 60°C, as described by Rakoczy et al. (2017). Staining was developed using BM-Purple Alkaline Phosphatase substrate (Sigma-Aldrich) and sections were counterstained using Nuclear Fast Red (Sigma-Aldrich). DIG-labelled sense riboprobes with identical sequences to the target mRNA were used as negative controls.

### Immunohistochemistry

Apoptosis and proliferation levels were measured by immunostaining in dewaxed and rehydrated midline placental sections with cleaved caspase-3 (Asp175) (Cell Signaling Technology, 9661; 1:200) and Ki67 (Abcam, ab264429; 1:500). Sections were incubated with goat-anti-rabbit secondary antibody (Abcam, ab6720; 1:1000), streptavidin-horseradish peroxidase (Rockland, S000-03, 1:250) and stained with 3,3′-diaminobenzidine (Abcam). Haematoxylin was used as a counterstain before dehydrating and mounting the sections. Caspase- and Ki67-positive cells were identified and counted in the placental Jz using NDP.view2 (Hamamatsu) (*n*=3-5 per genotype/sex, across five litters). Negative controls were prepared by the omission of primary antibodies.

### Glycogen assay

Amyloglucosidase was used to indirectly measure glycogen content in bisected placental halves (*n*=8 per genotype/sex, across eight litters), as previously described (Sferruzzi-Perri et al., 2013b). The concentration of glycogen was then extrapolated using total Jz weight to produce total placental glycogen content.

### Placental gene expression

Total RNA was extracted and 5 µg reverse transcribed from paired isolated Jz and Lz (*n*=8-10 per genotype/sex, across 11 litters) using the RNeasy Plus Mini Kit (Qiagen) and the High-Capacity cDNA Reverse Transcription Kit minus RT inhibitor (Applied Biosystems), according to the manufacturers’ instructions. Primer sequences were sourced from publications and reported previously (Aykroyd et al., 2020) or designed using NCBI Primer Blast and produced by Sigma-Aldrich (Table S2). Only primers which produced a PCR product of the desired size, correct sequence and with amplification efficiencies of >85% were used. Samples were measured in duplicate on a 7500 fast real-time PCR machine (Applied Biosystems) with MESA Blue SYBR (Eurogentec, BE) under the following conditions: 3 min at 95°C then 40 cycles of 30 s at 95°C, 30 s at 57°C, 90 s at 72°C. The cycle threshold expression values for each gene were normalised to the geometric mean of housekeeping genes *Hprt* and *Ywhaz* for Jz samples and *Hprt* and *Polr2a* for Lz samples. All reference genes were unaltered by genotype or sex. Fold change was calculated according to the 2^−ΔΔCT^ method (Livak and Schmittgen, 2001) and represents an estimation of fold change at the cellular level.

### Placental Jz protein expression

Protein was extracted from ∼50 mg of placental Jz tissue (*n*=7 per genotype/sex, across nine litters) using RIPA buffer (Thermo Fisher Scientific) containing cOmplete Mini EDTA-free protease inhibitor cocktail mix (Roche). The protein concentration of Jz lysates was determined using the Bicinchoninic Acid protein assay (Thermo Fisher Scientific). Lysates were diluted to 2.5 µg/µl in lysis buffer and 1× SDS, resolved using SDS-PAGE and transferred onto 0.2 μm nitrocellulose membranes (Bio-Rad Laboratories). Even protein loading and successful protein transfer was confirmed using Ponceau S stain (Sigma-Aldrich) before probing with primary antibodies (Table S3). Anti-rabbit secondary antibody tagged to horseradish peroxidase (NA934 Cytiva, 1:10,000 in 1× TBST with 2.5% milk/bovine serum albumin) was used for all membranes. Bands were visualised using Scientific SuperSignal West Femto enhanced chemiluminescence substrate (Thermo Fisher Scientific) and imaged using an iBright 1500 Imaging System (Invitrogen). Abundance of proteins was quantified using ImageJ analysis software (National Institutes of Health) to measure the pixel intensity of protein bands. Protein loading was controlled for by normalising against Ponceau S staining.

### Statistics

Before statistical analysis, a Prisms Grubbs’ test (GraphPad Software) was performed on all datasets to identify any outliers as a quality check. In the majority of cases, entire datasets did not contain outliers and, if they did, at most a single sample from a group was excluded. Final sample numbers are detailed within each table or figure legend. With the exception of protein abundance analyses, all data were analysed by two*-*way ANOVA (genotype and sex). If an overall significant effect of sex or genotype was identified, then planned comparisons using unpaired two-tailed *t*-tests were performed. Protein abundance was assessed for males and females separately to maintain a high sample size (*n*) per group and effect of genotype determined using unpaired two-tailed *t*-tests. Prism (GraphPad Software) was used to perform statistical analyses with a significance value of *P*<0.05. Results are shown as mean±s.e.m., *n*=number of fetuses or placentas in each group.

## Supplementary Material

Supplementary information

Reviewer comments
