# Supplementary material for: Loss of imprinting of the Igf2-H19 ICR1 enhances placental endocrine capacity via sex-specific alterations in signalling pathways in the mouse
Source: Development. 2022 Jan 4;149(1):dev199811. doi: 10.1242/dev.199811 (PMC8783045; doi:10.1242/dev.199811)
Supplement: Supplementary information [file develop-149-199811-s1.pdf]

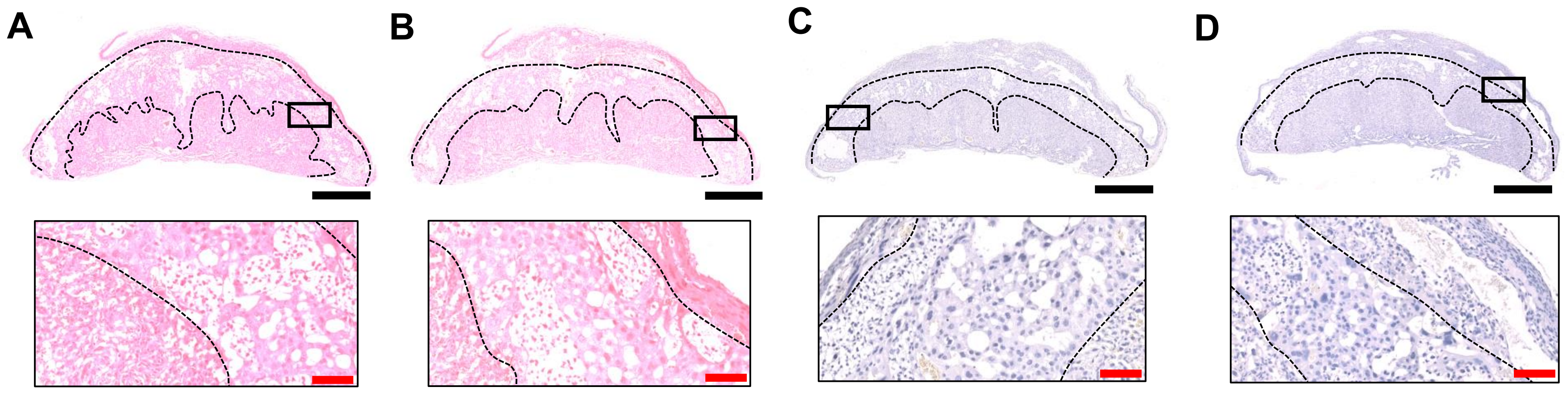

Fig. S1. Negative controls for *in situ* hybridization and immunohistochemistry experiments in D16 mouse placentas. Sense *in situ* hybridization probes for (A) *H19* and (B) *Psg23*. Secondary antibody only control used in immunohistochemistry experiments for (C) cleaved caspase-3 and (D) Ki67. Black boxes represent the area magnified in the image below. Black bar represents 1 mm. Red bar represents 100 µm.

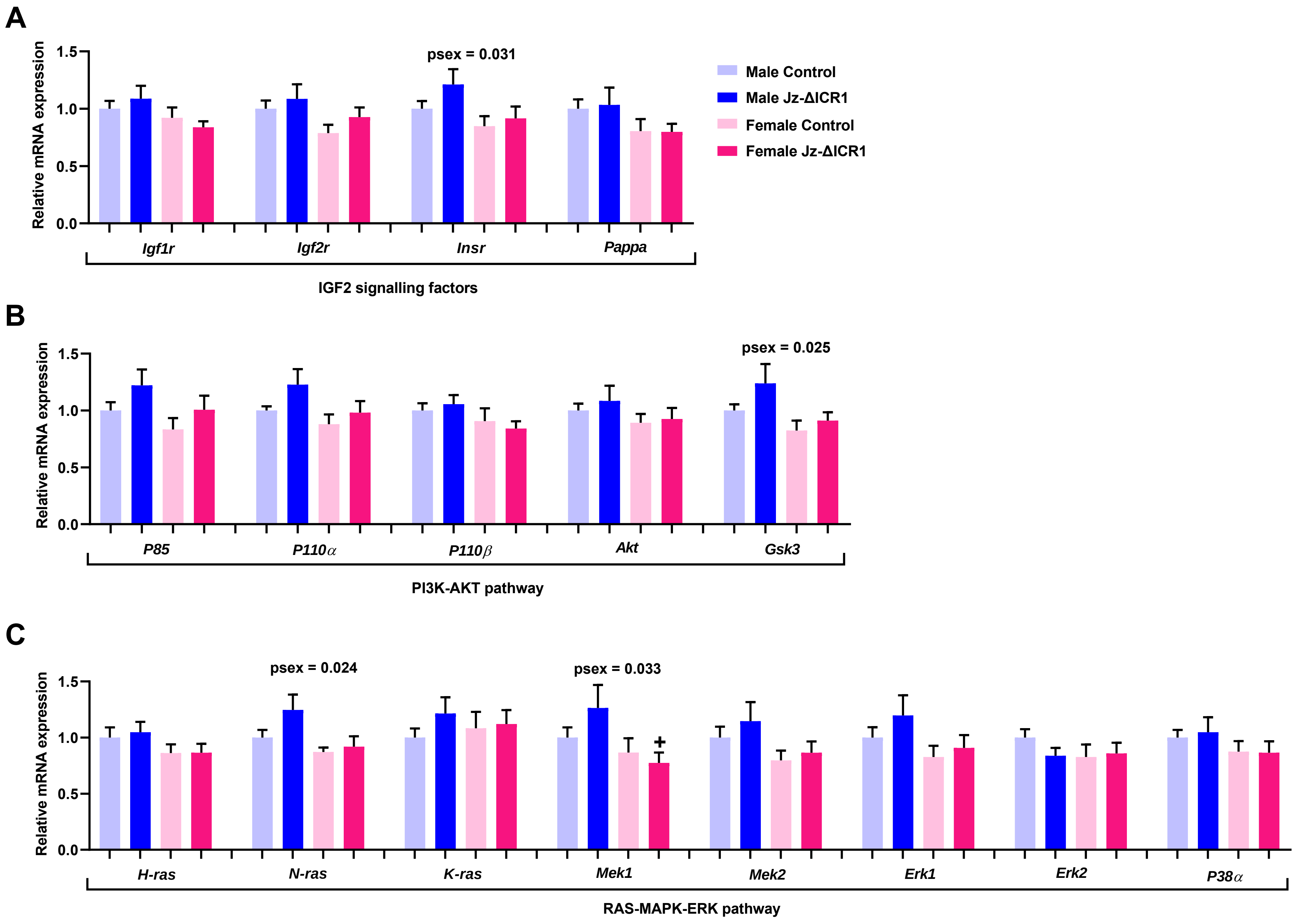

Fig. S2. Jz-ΔICR1 does not alter the gene expression of IGF2 signalling factors or downstream members of the PI3K-AKT and MAPK pathways. Expression of (A) IGF2 signalling factors, (B) PI3K-AKT pathway genes and (C) RAS-MAPK-ERK pathway genes in Jz samples relative to the geometric mean of housekeeping genes (*Hprt* and *Ywhaz*) using qPCR (n = 8-10 per genotype/sex, across 11 litters). Data were obtained on D16. Values presented as mean + SEM with significance assessed by two-way ANOVA and pairwise T-test (psex < 0.05 = +).

Development • Supplementary information

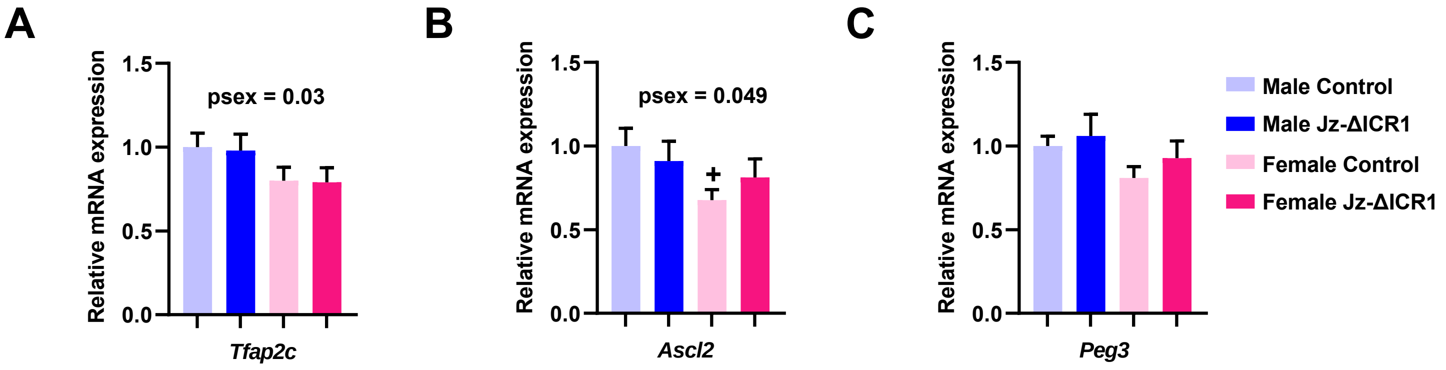

Fig. S3. Jz-ΔICR1 does not alter the gene expression of *Tfap2c, Ascl2* or *Peg3*. Expression of (A) Tfap2c, (B) *Ascl2* and (C) *Peg3* in Jz samples relative to the geometric mean of housekeeping genes (Hprt and Ywhaz) using qPCR (n = 9-10 per genotype/sex, across 11 litters). Data were obtained on D16. Values presented as mean + SEM with significance assessed by two-way ANOVA and pairwise T-test (psex < 0.05 = +).

Development • Supplementary information

Fig. S4.

A

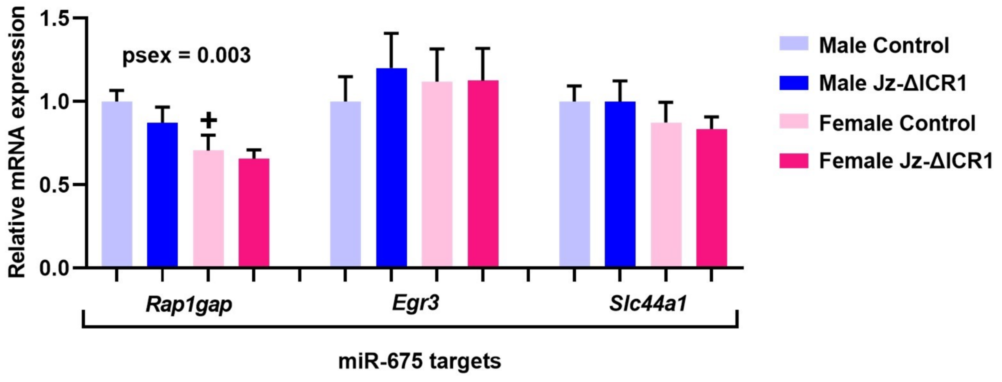

Fig. S4. Jz-ΔICR1 does not alter the gene expression of miR-675 targets. (A) Expression of miR-675 target genes in Jz samples relative to the geometric mean of housekeeping genes (*Hprt* and *Ywhaz*) using qPCR (n = 8-10 per genotype/sex, across 11 litters). Data were obtained on D16. Values presented as mean + SEM with significance assessed by two-way ANOVA and pairwise T-test (psex < 0.05 = +).

A

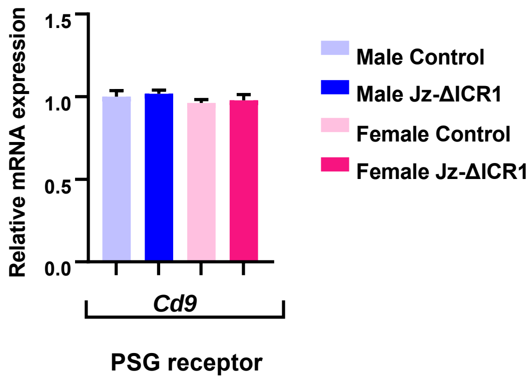

Fig. S5. Jz-ΔICR1 did not alter the expression of the PSG receptor *Cd9* in the Lz. (A) Expression *Cd9* in in Lz samples relative to the geometric mean of housekeeping genes (*Hprt* and *Polr2a*) with Jz-ICR1Δ using qPCR (n = 9-10 per genotype/sex, across 11 litters). Data were obtained on D16. Values presented as mean + SEM with significance assessed by two-way ANOVA.

Development • Supplementary information

Table S1. The effect of Jz-ΔICR1 on conceptus weight, placental volume and the proportion of cell types within the Jz and Db of D16 mouse placentas in males and females.

	Male		Female		Overall P Values		
	Control	Jz-ΔICR1	Control	Jz-ΔICR1	Genotype	Sex	Interaction
Fetal weight (mg)	420.4 ± 8.4	429.1 ± 11.8	407.5 ± 12.7	412.3 ± 14.2	0.6646	0.1667	0.7708
Placental weight (mg)	123.1 ± 4.0	120.7 ± 3.2	108.2 ± 4.6 †	110.8 ± 4.3	0.9560	0.0008	0.7987
Fetal:placental weight ratio	3.5 ± 0.1	3.5 ± 0.1	3.8 ± 0.2	3.8 ± 0.1	0.8413	0.0198	0.7029
Db volume (mm^3)	18.9 ± 1.1	14.3 ± 1.0 *	15.7 ± 0.5	15.5 ± 0.7	0.0104	0.2645	0.0679
Jz volume (mm^3)	45.0 ± 2.3	54.1 ± 2.9	33.9 ± 2.9 †	48.1 ± 1.7 *	<0.0001	0.0018	0.3148
Lz volume (mm^3)	52.1 ± 2.4	46.3 ± 1.9	47.4 ± 1.1	44.4 ± 1.3	0.0179	0.0680	0.4417
Jz SpT volume (mm^3)	27.5 ± 0.9	33.4 ± 1.2 *	22.3 ± 1.7 †	29.7 ± 1.2 *	<0.0001	0.0016	0.5768
Jz GC volume (mm^3)	15.8 ± 1.4	18.5 ± 1.8	10.6 ± 1.2	16 ± 1.0 *	0.0065	0.0091	0.3494
Jz TGC volume (mm^3)	1.8 ± 0.2	2.2 ± 0.3	1.0 ± 0.2	2.0 ± 0.2 *	0.0077	0.0649	0.2109
Db S volume (mm^3)	11.6 ± 0.6	8.5 ± 0.5 *	10.2 ± 0.6	11.1 ± 0.8 †	0.3655	0.0800	0.0034
Db GC volume (mm^3)	6.2 ± 0.5	5.0 ± 0.5	4.7 ± 0.2	4.8 ± 0.5	0.2235	0.0718	0.1724
Db V volume (mm^3)	1.0 ± 0.2	0.9 ± 0.2	0.8 ± 0.3	0.8 ± 0.2	0.5724	0.4757	0.7436
Jz GC / Jz SpT	0.57 ± 0.04	0.55 ± 0.04	0.48 ± 0.04	0.55 ± 0.04	0.5264	0.2328	0.2640
Db GC / Jz GC	0.42 ± 0.05	0.29 ± 0.02	0.49 ± 0.06	0.31 ± 0.04 *	0.0037	0.3990	0.6375
Db GC / (Jz GC + Db GC)	0.29 ± 0.03	0.22 ± 0.02	0.32 ± 0.03	0.23 ± 0.02 *	0.0044	0.4158	0.7247
Average Jz SpT cell size (μm^2)	308.3 ± 18.4	288.7 ± 17.8	305.5 ± 15.5	294.4 ± 20.5	0.4151	0.9379	0.8192
Average Jz GC cell size (μm^2)	211.6 ± 20.9	211.4 ± 14.8	174.2 ± 15.7	186.6 ± 18.7	0.7357	0.1041	0.7292
Average number of Jz SpT	91917.9 ± 7033.4	123267.1 ± 3010.1 *	70356.7 ± 9146.4	101629.7 ± 6729.3 *†	0.0006	0.0083	0.9957
Average number of Jz GC	76471.8 ± 7866.7	100092.8 ± 13213.5	60704.5 ± 7437.7	99236.4 ± 11529.4 *	0.0107	0.4356	0.4832

Weight data displayed for males (Control n = 29 and Jz-ΔICR1 n = 27, from individual pups across 13 litters) and females (Control n = 17 and Jz-ΔICR1 n = 27, from individual pups across 13 litters). Control:Jz-ΔICR1 male = 52:48 and female = Control:Jz-ΔICR1 = 39:61. Volume data: n = 8 per sex/genotype, across 7 litters. Cell size data: n = 4 per sex/genotype, across 4 litters. Values presented as mean ± SEM with significance assessed by two-way ANOVA and pairwise T-test (pgenotype < 0.05 = *, psex < 0.05 = †). Decidua (Db), Junctional zone (Jz), Labyrinth zone (Lz), Jz spongiotrophoblasts (Jz SpT), Jz glycogen cells (Jz GC), Jz trophoblast giant cell (Jz TGC), Db stroma (Db S), Db glycogen cell (Db GC) and Db vessel (Db V).

Table S2. QPCR primers with references for those previously published.

Gene	Forward	Reverse	Product size (bp)	Reference
Ascl2	GAGCAGGAGCTGCTTGACTT	CAGTCAGCACTTGGCATTTG	176	Tunster et al., 2010
Cd9	GCTGGGATTGTTCTTCGGGT	GGGTTCATCCTTGCTCCGTA	151	
Flt1	AAAGGCTGAGCATCACTCCC	GGACTCCCTGCATCACTAACAA	190	
Gbe1	GGACGCTGAGATGTACACGA	AGTCCAACCATTCAGGATGC	167	Tunster et al., 2010
Gys1	GGAGAAATTTGGGAGGAAGC	GTCAAGATGGGGTCTGAGGA	183	Tunster et al., 2010
H19	CATTCTAGGCTGGGGTCAAA	GCCCTTCTTTTCCATTCTCC	172	Keniry et al., 2012
Hsd11b1	GAGGAAGGTCTCCAGAAGGTA	ATGTCCAGTCCGCCCAT	143	Chin et al., 2017
Hsd11b2	GGCTGGATCGCGTTGTC	CGTGAAGCCCATGGCAT	132	Chin et al., 2017
Pappa	TGCAATAGGGTCAGAGTGTGC	GGGTATGTCACGCACTGTCA	91	
Pcdh12	GCCTGGTTAGGCTCTCTGTG	TTATTTCCTCGGTGGTTTGG	151	Tunster et al., 2010
Peg3	AGCACATCCCACTGTACGAA	TCTGCATTTGACCCCTGGAT	190	
Polr2a	CACTGTCATCACCCCTGACC	ATACTGGCTGTTTCCCCTGC	148	
Prl6a1	CTTACTGCCTGCCACACATC	CTCCAAGCTGTCAACATACTGA	114	
Prl7b1	CAGCACATCAATAGCCTTGC	TTGGTGATTTGAGTGGCAAA	160	Tunster et al., 2016
Rapgap1	CTGCAACTTCCGCAACAAGG	ATGTCGTTCCCGATATGCCG	108	
Slc2a1	GCTTATGGGCTTCTCCAAACT	GGTGACACCTCTCCCACATAC	123	Xu et al., 2015
Slc2a3	GATCGGCTCTTTCCAGTTTG	CAATCATGCCACCAACAGAG	176	Tunster et al., 2010
Slc38a1	CCTTCACAAGTACCAGAGCAC	GGCCAGCTCAAATAACGATGAT	127	Chen et al., 2015
Slc38a2	TAATCTGAGCAATGCGATTGTGG	AGATGGACGGAGTATAGCGAAAA	129	Chen et al., 2015
Slc38a4	GCGGGGACAGTATTCAGGAC	GGAACTTCTGACTTTCGGCAT	102	Chen et al., 2015
Slc44a1	TCCGCCGCGCAGAGC	TCTTGCTGCTGCACCTGTTG	147	
Tfap2c	CCTGCTCAGCTCCACGTC	CCTCCATTTTTGGACTTTGC	129	
Ugp2	CTGGTGGAAATCGCTCAAGT	TTACATTCAGGCCTCCATCC	187	Tunster et al., 2010
Vegfa	GAAGCTACTGCCGTCCGATT	CTTCATCGTTACAGCAGCC	126	

Table S3. Primary antibodies used for protein expression analysis

Primary antibody	Species	Manufacturer	Catalogue number	Dilution	Incubation time
Igf1r	Rabbit	Santa Cruz	sc-713	1:1000	1 O/N at 4°C, 2h at RT
Igf2r	Rabbit	Cell Signalling	15128	1:2000	1 O/N at 4°C, 2h at RT
Insr	Rabbit	Santa Cruz	sc-711	1:400	1 O/N at 4°C, 2h at RT
P85	Rabbit	Milipore	06-195	1:1000 in 5% BSA	2h at RT
P110α	Rabbit	Cell Signalling	4249	1:1000	1 O/N at 4°C, 2h at RT
P110β	Rabbit	Cell Signalling	3011	1:1000	1 O/N at 4°C, 3h at RT
pAkt Ser473	Rabbit	Cell Signalling	9271	1:500	1 O/N at 4°C, 3h at RT
T-Akt	Rabbit	Cell Signalling	9272	1:1000	1 O/N at 4°C, 2h at RT
pGsk3 Ser21/9	Rabbit	Cell Signalling	9331	1:1000	1 O/N at 4°C, 1h at RT
T-Gsk3	Rabbit	Cell Signalling	9315	1:1000	1 O/N at 4°C, 1h at RT
pP38	Rabbit	Cell Signalling	4511	1:1000	1 O/N at 4°C, 1h at RT
T-P38	Rabbit	Cell Signalling	8690	1:1000	1 O/N at 4°C, 1h at RT
pMapk Thr202/Tyr204 (Erk1/2)	Rabbit	Cell Signalling	4370	1:1000	1 O/N at 4°C, 2h at RT
T-Mapk (Erk1/2)	Rabbit	Cell Signalling	4695	1:1000	1 O/N at 4°C, 2h at RT

Abbreviations: O/N = overnight, RT = room temperature.

Supplementary reference list

Chen, S., Sun, F.Z., Huang, X., Wang, X., Tang, N., Zhu, B. and Li, B. (2015). Assisted reproduction causes placental maldevelopment and dysfunction linked to reduced fetal weight in mice. *Scientific reports.* **5**, 1-15.

Chin, E.H., Schmidt, K.L., Martel, K.M., Wong, C.K., Hamden, J.E., Gibson, W.T., Soma, K.K. and Christians, J.K. (2017). A maternal high-fat, high-sucrose diet has sex-specific effects on fetal glucocorticoids with little consequence for offspring metabolism and voluntary locomotor activity in mice. *PLoS One*. **12**, p.e0174030.

Tunster, S.J., Tycko, B. and John, R.M. (2010). The imprinted Phlda2 gene regulates extraembryonic energy stores. *Molecular and cellular biology*. **30**, 295-306.

Xu, W., Barrientos, T., Mao, L., Rockman, H.A., Sauve, A.A. and Andrews, N.C. (2015). Lethal cardiomyopathy in mice lacking transferrin receptor in the heart. *Cell reports*. **13**, 533-545

Development • Supplementary information
