# Supplementary material for: Loss of imprinting of the Igf2-H19 ICR1 enhances placental endocrine capacity via sex-specific alterations in signalling pathways in the mouse
Source: Development. 2022 Jan 4;149(1):dev199811. doi: 10.1242/dev.199811 (PMC8783045; doi:10.1242/dev.199811)
Supplement: Reviewer comments [file develop-149-199811_review_history.pdf]

Loss of imprinting of the *Igf2-H19* ICR1 enhances placental endocrine capacity via sex-specific alterations in signalling pathways in the mouse.

Bethany R. L. Aykroyd, Simon J. Tunster and Amanda N. Sferruzzi-Perri
DOI: 10.1242/dev.199811

Editor: Liz Robertson

Review timeline
Original submission: 17 May 2021
Editorial decision: 18 June 2021
First revision received: 8 November 2021
Accepted: 23 November 2021

Original submission

First decision letter

MS ID#: DEVELOP/2021/199811

MS TITLE: Loss of imprinting of the Igf2-H19 ICR1 enhances placental endocrine capacity via sex-specific alterations in signalling pathways in the mouse.

AUTHORS: Bethany R.L. Aykroyd, Simon J Tunster, and Amanda N Sferruzzi-Perri

I have now received all the referees reports on the above manuscript, and have reached a decision. The referees' comments are appended below, or you can access them online: please go to BenchPress and click on the 'Manuscripts with Decisions' queue in the Author Area.

The overall evaluation is positive and we would like to publish a revised manuscript in Development, provided that the referees' comments can be satisfactorily addressed. Please attend to all of the reviewers' comments in your revised manuscript and detail them in your point-by-point response. If you do not agree with any of their criticisms or suggestions explain clearly why this is so.

We are aware that you may currently be unable to access the lab to undertake experimental revisions. If it would be helpful, we encourage you to contact us to discuss your revision in greater detail. Please send us a point-by-point response indicating where you are able to address concerns raised (either experimentally or by changes to the text) and where you will not be able to do so within the normal timeframe of a revision. We will then provide further guidance. Please also note that we are happy to extend revision timeframes as necessary.

Reviewer 1

Advance summary and potential significance to field

In this manuscript, the authors showed the importance of the ICR1 in the placental endocrine function. The authors utilized a novel mouse model called Jz-ΔICR1, where the ICR1 is selectively deleted in the endocrine junctional zone of the placenta. The mouse model showed increased Igf2 and decreased H19 expression together with increase of the endocrine cell types in the junctional

© 2022. Published by The Company of Biologists under the terms of the Creative Commons Attribution License (https://creativecommons.org/licenses/by/4.0/).

zone and increased expression of pregnancy-specific glycoprotein 23. The highlight of this study is the sexually dimorphic alterations related to the IGF2 receptors and the downstream signaling pathways.

Comments for the author

The paper was very well written, introduction was excellent and had a good outline for the different experiments they performed. Nevertheless, there are a few comments, possible edits that could enhance their work.

- Did the authors observe changes in sex ratio and/or litter size under Jz-ΔICR1? Is there a difference in survival between the sexes? Are the changes observed litter specific? Are the same females and males affected in the whole litter? Or just a few?
- Was Fetal to Placental weight ratio affected by Jz-ΔICR1, especially in sex-specific manner?
- Regarding the molecular analysis of placental tissue, how did the authors rule out the possibility of maternal contamination?
- In line 201-206, the authors mentioned that Jz volume in the placenta was significantly lower in females compared to males in the control group. Do the authors have possible explanations for this phenomenon?

Could this be because the mother would be compensating for the lower endocrine function in female pups?

- In line 220-224, the authors mention an interaction between genotype and sex in determining the total volume of decidual stroma. Figure 3K showed these results, but the authors never stated that there are no differences with control females.
- For all the immunohistochemistry, it would be helpful to add arrows or guides to indicate the positive cell marks. Additionally, please describe how the data were normalized (e.g. by cell number or by total placental area).
- In figure 4D, the number of Caspase-3 positive cells is highly variable in all groups. Could the authors measure other apoptotic markers, such as Bax or Bcl2?
- In line 235 – 243, from the Fig. 5A, the expression of Prl8a8 and Gjb3 appears to be lower in the "Female Jz-ΔICR1" group, although it is not statistically significant. Would it be significant if "Female Jz-ΔICR1" group is compared only to "Female Control" group? In statistical analysis, would it be reasonable to treat males and females as independent groups, instead of grouping both sexes?
- In line 246, the authors mentioned that the increase of the total placental glycogen in Jz-ΔICR1 was significant in females when data were separated by sex. This statement is inaccurate since they never showed the data together.
- With respect to the introduction and discussion, the authors keep referring to the genes Ascl2, Peg3 and Phlda2. Did the authors measure the expression of these genes in the placenta of their mouse model? It would be interesting to determine the levels of three major drivers of placental endocrine function in this mouse model and see possible influence of the Igf2-H19 pathway.
- Were any changes observed in the fetuses of this mice? Is there any evident alteration in growth or development due to this overexpression of Igf2? Are these fetuses viable? Is there any pathway that can compensate or rescue the strong phenotype the authors observed?
- The authors mention that some other pathways/mechanisms controlling the placental gene expression could be involved. Did the authors examine if the epigenetic regulation such as DNA methylation, DNMT expression or histone posttranslational modifications were affected by Jz-ΔICR1? Could one of these mechanisms compensate the lack of ICR1?
- It is unclear if the authors verified their various differences in terms of percentage of placenta. Also, how did the authors choose which placenta to analyze? Were all collected placentas analyzed?
- Line 357-368; Could the changes in glycogen deposition be a compensatory effect of the placenta due to the lack of glycogen reserved during growth? Is this increase in glycogen present since early placental formation? If so, did the authors analyze earlier time points?
- Line 383 – 387; the expression of Flt1 was not affected by Jz-ΔICR1. However, the level of sFlt1, which is the active form of Flt1 in the VEGF pathway, was not shown. Also, the authors mentioned artery remodeling which changes the labyrinth villi. This is very interesting, did the authors observe changes in the number of microvessels or the level of endothelial cells?

© 2022. Published by The Company of Biologists under the terms of the Creative Commons Attribution License (https://creativecommons.org/licenses/by/4.0/).

Major comments:
• In Figure 2B and C, H19/Igf2 expression in SpT was not affected or minimally affected by Jz-ΔICR1. Still SpT was the only population for which volume was increased by Jz-ΔICR1. Could this be because of the circulating IGF2 affecting SpT? In that case, would this be due to paracrine IGF2 that GC secretes? Could the authors measure the placental (or in Jz) IGF2 concentration in control vs. Jz-ΔICR1?
• Related to the earlier point, was expression of IGF2 receptors altered in SpT population of Jz-ΔICR1 placenta (is IHC available for the receptors)? Figure 7 addresses the altered protein level of these receptors in the entire Jz, not the specific cell type. This might suggest a reason why SpT was more affected compared to other cell types.
• In Figure 5D, the "total placental glycogen content" is not from the whole placenta. According to the methods section, this value was measured using the half of each placenta. In this case, there's no guarantee that this is the accurate representation of the whole placental glycogen content, since there might be variability in each placenta, according to the cut. It seems more logical to normalize this to the total weight of the analyzed placenta, which was shown in Figure 5C and was not statistically significant. It is challenging to conclude that the placental glycogen store was affected just based on Figure 5D when Figure 5C and 5E (PAS staining) show that there's no significant difference in glycogen storage %. Or PAS staining in 5E could be quantified?
• Regarding the sexual dimorphism in the signaling pathways affected by Jz-ΔICR1, could it be related to the different sex hormones in placenta? Any information of sex hormone expression/concentration in placenta at/before embryonic day 16?

Minor points
• In Fig. 4D, the number of caspase-3 positive GC seems to be affected by Jz-ΔICR.
• In Fig. 5F, the Tpbpa signal in female placenta looks more intense in control compared to the one in Jz-ΔICR. Is this just the technical variability, or was Tpbpa expression significantly higher in the control female placenta?

Reviewer 2

Advance summary and potential significance to field

Overall, this is highly interesting and well written manuscript that was easy to follow. The use of a mouse model to selectively remove the ICR1 from the mouse JZ to investigate it's role in placental formation is novel and highly informative. It highlights that deletion of ICR1, increases IFG2 while decreasing H19 expression within the JZ. This allows for further interrogation of these proteins in placental function. The resulting phenotype was highly interesting as alterations to the JZ cell populations are commonly reported in animal models of pregnancy dysfunction. While all three cell types increased in abundance within this region, it was clear that this was due to enhanced proliferation rather than increased cell size. Given such macroscopic changes to this important endocrine region, it was surprising that quite minimal changes to the expression of placental peptides were reported. Interestingly, this study did show that a novel peptide that is poorly understood was increased by a significant margin. Importantly, this study also highlights that fetal sex plays a key role in regulating growth of the placenta in the context of an important imprinted genetic region. Overall, the change in physiology is highly i nteresting and highlights the importance of this system to placental growth and f unction.

Comments for the author

I would encourage revision based on the relatively minor comments below.
1- I was surprised that given that the authors highlight that the role of ICR1 in nutrient allocation to the fetus is unknown, that nutrient concentrations in the fetus were not measured. It would have been quite interesting to measure fetal glucose concentrations particularly in light of the altered glycogen content in these placentas. Perhaps, the increase in glycogen has occurred at the expense of glucose transport to the fetus or glucose usage by the placenta for cellular function and growth. Do you have this data available and could it be included if you do have this data? If not, could you please speculate in the discussion.

© 2022. Published by The Company of Biologists under the terms of the Creative Commons Attribution License (https://creativecommons.org/licenses/by/4.0/).

2- While Slc2a1 was not impacted in the JZ, there might have been some indirect effects of changes in JZ glycogen content and endocrine function on labyrinth transport. Can you comment on how these impacts might have affected the labyrinth?
3-One of your major findings was that psg23 expression was increased. It would have been great to see protein abundance of this gene but understand that there are unlikely to be any antibodies available to test this. You have alluded to the fact that changes to this gene may impact other aspects of placental function that have not been measured in this study but it is not clear why this gene was increased in the JZ while other peptides were not. Could you comment on this further in the discussion?
4-You make a good point in the discussion about the potential impact of changes in the endocrine function of the placenta on maternal physiology. This in turn may impact the development of wild type littermates. Can you indicate if the placental weights and fetal weights of the wild type littermates in this model are similar to placental/fetal weights from this gestational age from previous studies?
5- You highlight that cells with the 100um2 size range are increased. Can you please be specific in the methods section as to how this range is assessed (ie.
is this cells between 50 and 150?)
6- How do the GC cells in the decidua differ from those in the JZ. Do these share similar lineage? Are they cells which have migrated and if so why would they not be impacted in this model? Can this be made clear in the manuscript.
7-Why would the expression of cell markers not change if the stereology has demonstrated a change in the composition of the JZ? Would this be due to the fact that entire JZ increased in size and that each of the cell types likely contributed to this increase in JZ?
It would appear that the stereology was performed on a smaller sample size using visual inspection which may be less accurate than using genetic markers that are known to correlate with abundance of a specific cell type. Could you comment further on the difference on outcomes based on gene expression compared to stereology.
Minor comments Line 550- Please indicate the number of fetuses used per litter.

First revision

Author response to reviewers' comments

Reviewer 1 Advance Summary and Potential Significance to Field:
In this manuscript, the authors showed the importance of the ICR1 in the placental endocrine function. The authors utilized a novel mouse model called Jz-ΔICR1, where the ICR1 is selectively deleted in the endocrine junctional zone of the placenta. The mouse model showed increased Igf2 and decreased H19 expression, together with increase of the endocrine cell types in the junctional zone and increased expression of pregnancy-specific glycoprotein 23. The highlight of this study is the sexually dimorphic alterations related to the IGF2 receptors and the downstream signaling pathways.

Reviewer 1 Comments for the Author:
The paper was very well written, introduction was excellent and had a good outline for the different experiments they performed. Nevertheless, there are a few comments, possible edits that could enhance their work.

- Did the authors observe changes in sex ratio and/or litter size under Jz-ΔICR1? Is there a difference in survival between the sexes?

In general, we did not observe a change in the expected sex ratio, genotype frequency and size of litters containing Jz-ΔICR1 conceptuses. However, please note that our study was performed on litters containing both control and Jz-ΔICR1 conceptuses (by mating homozygous TpbpaCre males with heterozygous ICR floxed females), which was done to control for any potential differences in the maternal environment. These data have now been included in the revised paper, please see lines 185- 188.

© 2022. Published by The Company of Biologists under the terms of the Creative Commons Attribution License (https://creativecommons.org/licenses/by/4.0/).

- Are the changes observed litter specific? Are the same females and males affected in the whole litter? Or just a few?

No, changes observed were not litter specific. All individuals were included in fetal and placental weight analysis and therefore represent the changes occurring for the males and females in the whole litter. Placentae with weights closest to the mean value for each sex and genotype within each litter were selected for detailed structural and molecular analysis.

- Was Fetal to Placental weight ratio affected by Jz-ΔICR1, especially in sex-specific manner?

Fetal to Placental weight ratio was unaffected by genotype. However, regardless of genotype, males had a lower fetal:placental weight ratio when compared with females. We have included these data in the revised paper (See Supp Table 1 and text in lines 202-204 and 551).

- Regarding the molecular analysis of placental tissue, how did the authors rule out the possibility of maternal contamination?

The molecular analysis of placental tissue was conducted on junctional zones separated from the labyrinthine zone that contains maternal blood which is considered the greatest source of maternal contamination. Although the separated junctional zone contains maternal decidua, it is considered part of the mouse placenta (even if it is only a very small part of it), and thus it is important that this region is included in such phenotypic assessments. For instance, glycogen cells migrate into the decidua, and excluding this region (say by using laser capture microdissection on placental sections) would not contain all glycogen cell populations.

- In line 201-206, the authors mentioned that Jz volume in the placenta was significantly lower in females compared to males in the control group. Do the authors have possible explanations for this phenomenon? Could this be because the mother would be compensating for the lower endocrine function in female pups?

A number of previous studies in wildtype mice have reported that the placenta of female fetuses is lighter than that of male fetuses (see lines 546-547 with two additional references to support this; Eriksson et al., 2010; Kalisch-Smith et al., 2017). Our data suggests that this is likely related to smaller Jz size and is accompanied by reduced Jz cell marker and hormone genes. We don't know if the mother may initiate a specific response to the lower placental endocrine function in females, but this may be predicted to affect all littermates equally, irrespective of sex. Recent published work in the early human placenta showed an enrichment of pathways essential for protein synthesis, cell growth and energy metabolism in males compared to females and these were largely linked to genes encoded by the X or Y chromosome (Gonzalez et al., 2018). We therefore suggest that our findings are likely mediated by the effect of fetal sex on placental formation and functional capacity. We have inserted text reinforcing this possible mechanism in the revised discussion, lines 552-561.

- Eriksson JG, Kajantie E, Osmond C, Thornburg K, Barker DJ. Boys live dangerously in the womb. American Journal of Human Biology. 2010 May;22(3):330-5.
- Kalisch-Smith JI, Simmons DG, Pantaleon M, Moritz KM. Sex differences in rat placental development: from pre-implantation to late gestation. Biology of sex differences. 2017 Dec;8(1):1-3.
- Gonzalez TL, Sun T, Koeppel AF, Lee B, Wang ET, Farber CR, Rich SS, Sundheimer LW, Buttle RA, Chen YD, Rotter JI. Sex differences in the late first trimester human placenta transcriptome. Biology of sex differences. 2018 Dec;9(1):1-23.

- In line 220-224, the authors mention an interaction between genotype and sex in determining the total volume of decidual stroma. Figure 3K showed these results, but the authors never stated that there are no differences with control females.

Thank you for pointing this out. We have corrected the statement on lines 224-225 to include "and not in control males compared to control females".

- For all the immunohistochemistry, it would be helpful to add arrows or guides to indicate the positive cell marks. Additionally, please describe how the data were normalized (e.g. by cell number or by total placental area).

© 2022. Published by The Company of Biologists under the terms of the Creative Commons Attribution License (https://creativecommons.org/licenses/by/4.0/).

We thank the reviewer for their suggestions. We have included arrows to indicate the positive cells in the images of the immunohistochemistry staining (Fig. 4). We have also included a description on how data were normalised in the corresponding figure legend.

- In figure 4D, the number of Caspase-3 positive cells is highly variable in all groups. Could the authors measure other apoptotic markers, such as Bax or Bcl2?

Previous studies have reported very low levels of apoptosis (~1%) in the placenta, and particularly the Jz at similar stages (Duval et al., 2017). In support of this, we only counted on average 3 or 4 positive cells per section for the entire placental Jz regardless of fetal sex and genotype. At such low frequency, a relatively small difference in the number of apoptotic cells observed will have contributed to greater variation in the data, as observed. Given that caspase-3 is barely detectable in the placental Jz and it is downstream of Bax or Bcl2 we feel that measuring Bax or Bcl2 would not provide any further information and may instead simply add more variable data to the paper.

• Duval C, Dilworth MR, Tunster SJ, Kimber SJ, Glazier JD. PTHrP is essential for normal morphogenetic and functional development of the murine placenta. Developmental biology. 2017 Oct 15;430(2):325-36.

- In line 235 – 243, from the Fig. 5A, the expression of Prl8a8 and Gjb3 appears to be lower in the "Female Jz-ΔICR1" group, although it is not statistically significant. Would it be significant if "Female Jz-ΔICR1" group is compared only to "Female Control" group? In statistical analysis, would it be reasonable to treat males and females as independent groups, instead of grouping both sexes?

We agree that an alternative method of analysis would be to undertake entirely separate analyses for males and females throughout. However, from the outset we were interested not only in characterising the phenotype associated with Jz-ΔICR1, but also to identify sexually dimorphic responses. In order to achieve this, we were obliged to first perform a Two-Way ANOVA to identify main effects (sex, genotype or interaction). Only if any main effects were identified were we able to subsequently undertake separate post-hoc analyses. In the case of *Prl8a8* and *Gjb3*, no main effects were identified by the Two-Way ANOVA, and thus we were not permitted to perform post hoc tests. Whilst this is a more conservative approach, it allows us to explore the effect of both genotype and sex in our model.

- In line 246, the authors mentioned that the increase of the total placental glycogen in Jz-ΔICR1 was significant in females when data were separated by sex. This statement is inaccurate since they never showed the data together.

Whilst we do not present combined data for any of our analyses, our statistical analyses first applies a Two-Way ANOVA to identify whether any of the main effects (sex, genotype or interaction) reach significance. In this case, we identified that there was an overall effect of genotype (Fig. 5D), however post-hoc analyses demonstrate that this is attributable to an effect only in females. We have rephrased this sentence to clarify its meaning (please see lines 245-248).

"Whilst placental glycogen concentration was not altered by Jz-ΔICR1 (Fig. 5C), total placental glycogen content was increased by Jz-ΔICR1. However, post-hoc analyses demonstrate that this is attributable to a +34% increase in the placenta of Jz-ΔICR1 females only (Fig. 5D)."

- With respect to the introduction and discussion, the authors keep referring to the genes Ascl2, Peg3 and Phlda2. Did the authors measure the expression of these genes in the placenta of their mouse model? It would be interesting to determine the levels of three major drivers of placental endocrine function in this mouse model and see possible influence of the Igf2-H19 pathway.

The overwhelming majority of mouse models with placental phenotypes focus their characterisation on placental nutrient transport and largely neglect an assessment of endocrine function. We refer to the Ascl2, Peg3 and Phlda2 models as examples of studies that have instead focused on the endocrine function of the placenta. To assess the involvement of such major endocrine genes in our model and as requested by the reviewer, we assessed the expression of

© 2022. Published by The Company of Biologists under the terms of the Creative Commons Attribution License (https://creativecommons.org/licenses/by/4.0/).

Ascl2 and *Peg3* by the placental Jz by qPCR. Both of these genes showed robust expression in the placental Jz, but neither were significantly altered at D16 by Jz-ΔICR1 (*Ascl2* expression was significantly decreased in the Jz of control females compared with control males). These data are included in the revised paper (see Fig. S3B,C and lines 294-297). We did not measure *Phlda2* as its expression in the placenta declines dramatically during gestation and is restricted to a small percentage of cells in the placental transport labyrinth zone (Lz) by D14.5 (Frank et al., 2002).

• Frank D, Fortino W, Clark L, Musalo R, Wang W, Saxena A, Li CM, Reik W, Ludwig T, Tycko B. Placental overgrowth in mice lacking the imprinted gene Ipl. Proceedings of the National Academy of Sciences. 2002 May 28;99(11):7490-5.

- Were any changes observed in the fetuses of this mice? Is there any evident alteration in growth or development due to this overexpression of Igf2? Are these fetuses viable? Is there any pathway that can compensate or rescue the strong phenotype the authors observed?

Fetal weight and viability were unaffected by Jz-ΔICR1 on D16, even though our data show that placental endocrine capacity is enhanced and may be expected to have positive effects on fetal development. In response to reviewer 2 comment 2, we assessed the expression of specific nutrient transporter genes in the placental Lz to see if the impacts of Jz-ΔICR1 on placental endocrine function may have a 'knock-on' effect on placental transport capacity. This analysis revealed that placental Lz glucose transporter expression (*Slc2a3*) was reduced on D16 for fetuses with Jz-ΔICR1. We suspect that this down-regulation of placental glucose transfer capacity may reflect an attempt to compensate for the increased placental endocrine capacity and least partly explain why fetal development is not enhanced with Jz-ΔICR1. However, we do not know whether there may be an effect on fetal growth in later gestation, for example on D19 when fetal growth rate is highest. We also do not know whether there may be an effect on fetal development if the entire litter is supported by Jz-ΔICR1. We have inserted sentences into the revised discussion about this (see lines 512-533 and 571-572).

- The authors mention that some other pathways/mechanisms controlling the placental gene expression could be involved. Did the authors examine if the epigenetic regulation such as DNA methylation, DNMT expression or histone posttranslational modifications were affected by Jz- ΔICR1? Could one of these mechanisms compensate the lack of ICR1?

We thank the reviewer for their comment. We cannot rule out that ΔICR1 may result in allele-specific DNA methylation and histone modification differences that may contribute to the observed phenotype. However, this is outside of the scope of our study. We have included a sentence in the discussion to acknowledge that epigenetic mechanisms may be altered by the lack of ICR1 and could be addressed in future work (lines 344-346).

- It is unclear if the authors verified their various differences in terms of percentage of placenta. Also, how did the authors choose which placenta to analyze? Were all collected placentas analyzed?

All conceptuses were included for fetal and placental weight analysis. However, for morphological and molecular analyses, a single placenta per litter for each of the four possible sex and genotype combinations (male control; male Jz-ΔICR; female control; female Jz-ΔICR) was selected as far as possible. Where multiple placentas of the same sex and genotype combination were present in the same litter, the placenta with the weight closest to the litter mean for that combination was selected. Based on the four genotype and sex combinations, and an average litter size of 8, our morphological and molecular analyses encompassed approximately 50% of the placentas generated. We have added additional clarification between lines 611-615.

- Line 357-368; Could the changes in glycogen deposition be a compensatory effect of the placenta due to the lack of glycogen reserved during growth? Is this increase in glycogen present since early placental formation? If so, did the authors analyze earlier time points?

Our study focused on undertaking a detailed characterisation on a single time point. Whilst glycogen concentration was unaltered, total placental glycogen content was increased. This

© 2022. Published by The Company of Biologists under the terms of the Creative Commons Attribution License (https://creativecommons.org/licenses/by/4.0/).

suggests that rather than representing a compensatory mechanism, the increased glycogen content simply reflects the increased Jz size. We have clarified this in our discussion and as glycogen cells accumulate glycogen from D12 of gestation, we have added that analysing different time points could be an interesting future direction (lines 383-385)

- Line 383 – 387; the expression of Flt1 was not affected by Jz-ΔICR1. However, the level of sFlt1, which is the active form of Flt1 in the VEGF pathway, was not shown. Also, the authors mentioned artery remodeling, which changes the labyrinth villi. This is very interesting, did the authors observe changes in the number of microvessels or the level of endothelial cells?

Measuring sFLT1 in maternal plasma would not be helpful given the dams carry litters of mixed genotype (control and Jz-ΔICR1 conceptuses). We aim to follow up on the phenotypic consequences of possible alterations to circulating hormones in models where the whole litter is control or Jz-ΔICR1.

We we did not see a change in Db vessels using stereology (Supplementary Table 1). However, we have suggested that future work should assess if there are changes in the morphology of the Lz (please see line 409).

Major comments:

- In Figure 2B and C, H19/Igf2 expression in SpT was not affected or minimally affected by Jz-△ICR1. Still, SpT was the only population for which volume was increased by Jz-△ICR1. Could this be because of the circulating IGF2 affecting SpT? In that case, would this be due to paracrine IGF2 that GC secretes? Could the authors measure the placental (or in Jz) IGF2 concentration in control vs. Jz-△ICR1?

We would like to urge caution in attempting to quantify gene expression from in situ hybridisation staining. The purpose of the in situs was solely to verify that spatial expression of Igf2 and H19 was not affected by Jz-△ICR1. Quantification of *Igf2* and *H19* expression was performed by qPCR, and demonstrated a ~30% increase in *Igf2* and a comparable decrease in *H19* in the Jz. Such differences in are unlikely to be obvious in the in situ hybridisation staining. However, the in suits do indeed show that *Igf2* and *H19* are highly expressed within the GCs of the Jz, with much lower (*Igf2*) or no/negligible (*H19*) expression detected in the SpT and TGC cells. Despite the more restricted/confined expression of Igf2 to the GCs, Jz-ΔICR1 increased the volume of all three Jz cell types (not just the SpT; please see Supplementary Table S1). We agree with the reviewer that effects on the other Jz cell types could be mediated by paracrine IGF2 from the GCs. However, as the *Igf2* gene is differentially expression by different Jz cell types, we don't believe measuring IGF2 protein concentrations, even in isolated Jzs would provide more information, especially in regard to putative paracrine signalling. We have highlighted putative role of IGF2 in paracrine signalling between cellular compartments of the placenta in the revised discussion. See lines 354-358.

- Related to the earlier point, was expression of IGF2 receptors altered in SpT population of Jz-△ICR1 placenta (is IHC available for the receptors)? Figure 7 addresses the altered protein level of these receptors in the entire Jz, not the specific cell type. This might suggest a reason why SpT was more affected compared to other cell types.

We thank the reviewer for their excellent point. IGF1R and IGF2R are localised throughout the Jz of the mouse placenta (Charnock et al., 2016). Therefore, there is potential for paracrine communication, but this would be difficult to assess this using IHC. Single cell mass spectrometry could be employed to assess protein levels in individual cell types, but this was out of scope for this study.

- Charnock JC, Dilworth MR, Aplin JD, Sibley CP, Westwood M, Crocker IP. The impact of a human IGF-II analog ([Leu27] IGF-II) on fetal growth in a mouse model of fetal growth restriction. American Journal of Physiology-Endocrinology and Metabolism. 2016 Jan 1;310(1):E24-31.

© 2022. Published by The Company of Biologists under the terms of the Creative Commons Attribution License (https://creativecommons.org/licenses/by/4.0/).

- In Figure 5D, the "total placental glycogen content" is not from the whole placenta. According to the methods section, this value was measured using the half of each placenta. In this case, there's no guarantee that this is the accurate representation of the whole placental glycogen content, since there might be variability in each placenta, according to the cut. It seems more logical to normalize this to the total weight of the analyzed placenta, which was shown in Figure 5C and was not statistically significant. It is challenging to conclude that the placental glycogen store was affected just based on Figure 5D when Figure 5C and 5E (PAS staining) show that there's no significant difference in glycogen storage %. Or PAS staining in 5E could be quantified?

By bisecting each placenta, we were able to maximise the number of analyses that would be performed and thus reduce our animal usage (3Rs), and is consistent with our previous approach (Aykroyd et al., 2020). The weight of tissue used for glycogen extraction was noted, glycogen content determined, and whole placental glycogen content and concentration extrapolated using total Jz weight to yield the data presented in Fig 5C and D. Further details have been added to the materials and methods section (lines 666-667).

The data that are presented in Figures 5C, 5D and 5E are subtly different. Figure 5C depicts placental glycogen concentration – this is unaltered and suggests that the amount of glycogen stored by each cell is unaffected. Fig 5D shows total placental glycogen content, which is slightly increased, and can be attributed to the increased number of glycogen cells in Jz-∆ICR1 placentas. Fig 5E can only be considered a semi-quantitative measure and is intended only as an evaluation of glycogen cell localisation. For more reliable quantification, the area and intensity of the PAS staining should be determined in multiple sections throughout the placenta. However due to its semi-quantitative nature, only a representative midline placental section was collected for PAS staining in our study.

- Aykroyd BR, Tunster SJ, Sferruzzi-Perri AN. Igf2 deletion alters mouse placenta endocrine capacity in a sexually dimorphic manner. Journal of Endocrinology. 2020 Jul 1;246(1):93-108.

- Regarding the sexual dimorphism in the signaling pathways affected by Jz-∆ICR1, could it be related to the different sex hormones in placenta? Any information of sex hormone expression/concentration in placenta at/before embryonic day 16?

We do not have any information on sex hormone concentration in the placenta, but expression of key genes in the steroidogenic pathway in the Jz were not affected by Jz-∆ICR1 at D16 (there was however a greater expression of *Stard1*, which is early in the steroidogenic pathway, in males versus females overall; Figure 6A). However, sex hormones from the fetal gonads and adrenal glands are produced from D10-13.5 in mice and these vary between male and females (Kalisch-Smith et al., 2017). Genetic factors, namely X and Y-linked genes are likely to also be involved (Ben-Haroush et al., 2012). In addition, some genes such as *Slc38a5* (an amino acid transporter) have been found to escape X inactivation with resultant impacts on gene dosage (Finn et al., 2014). Additionally, the Y chromosome contains coding genes (Gubbay et al., 1990; Yamauchi et al., 2014), which may influence placenta function and account for the observed sexual dimorphism with Jz-∆ICR1. But considerable further work is required to first elucidate the causal mechanisms underlying why male wildtypes are different to female wildtypes, before we will be able to better understand sex differences in mutant lines.

- Kalisch-Smith, J.I., Simmons, D.G., Dickinson, H. and Moritz, K.M., 2017. Sexual dimorphism in the formation, function and adaptation of the placenta. Placenta, 54, pp.10-16.
- Ben-Haroush, A., Melamed, N., Oron, G., Meizner, I., Fisch, B. and Glezerman, M., 2012. Early first-trimester crown-rump length measurements in male and female singleton fetuses in IVF pregnancies. The Journal of Maternal-Fetal & Neonatal Medicine, 25(12), pp.2610-2612.
- Finn, E.H., Smith, C.L., Rodriguez, J., Sidow, A. and Baker, J.C., 2014. Maternal bias and escape from X chromosome imprinting in the midgestation mouse placenta. Developmental biology, 390(1), pp.80-92.
- Gubbay, J., Collignon, J., Koopman, P., Capel, B., Economou, A., Münsterberg, A., Vivian, N., Goodfellow, P. and Lovell-Badge, R., 1990. A gene mapping to the sex-

© 2022. Published by The Company of Biologists under the terms of the Creative Commons Attribution License (https://creativecommons.org/licenses/by/4.0/).

determining region of the mouse Y chromosome is a member of a novel family of embryonically expressed genes. Nature, 346(6281), pp.245-250.

- Yamauchi, Y., Riel, J.M., Stoytcheva, Z. and Ward, M.A., 2014. Two Y genes can replace the entire Y chromosome for assisted reproduction in the mouse. Science, 343(6166), pp.69-72.

Minor points
- In Fig. 4D, the number of caspase-3 positive GC seems to be affected by Jz-∆ICR.

Whilst there appears to be a trend, given the very low baseline level of apoptosis, we are reluctant to speculate as to whether a change from an average of 1 to 0.5 positive GC cells in placental Jz midline sections has any biological significance.

- In Fig. 5F, the Tpbpa signal in female placenta looks more intense in control compared to the one in Jz-∆ICR. Is this just the technical variability, or was Tpbpa expression significantly higher in the control female placenta?

We thank the reviewer for their observation. However, as mentioned above, ISH is not quantitative and is only for spatial assessment.

Reviewer 2 Advance Summary and Potential Significance to Field:

Overall, this is highly interesting and well written manuscript that was easy to follow. The use of a mouse model to selectively remove the ICR1 from the mouse JZ to investigate it's role in placental formation is novel and highly informative. It highlights that deletion of ICR1, increases IFG2 while decreasing H19 expression within the JZ. This allows for further interrogation of these proteins in placental function. The resulting phenotype was highly interesting as alterations to the JZ cell populations are commonly reported in animal models of pregnancy dysfunction. While all three cell types increased in abundance within this region, it was clear that this was due to enhanced proliferation rather than increased cell size. Given such macroscopic changes to this important endocrine region, it was surprising that quite minimal changes to the expression of placental peptides were reported. Interestingly, this study did show that a novel peptide that is poorly understood was increased by a significant margin. Importantly, this study also highlights that fetal sex plays a key role in regulating growth of the placenta in the context of an important imprinted genetic region. Overall, the change in physiology is highly interesting and highlights the importance of this system to placental growth and function.

Reviewer 2 Comments for the Author:
I would encourage revision based on the relatively minor comments below.

1 I was surprised that given that the authors highlight that the role of ICR1 in nutrient allocation to the fetus is unknown, that nutrient concentrations in the fetus were not measured. It would have been quite interesting to measure fetal glucose concentrations particularly in light of the altered glycogen content in these placentas. Perhaps, the increase in glycogen has occurred at the expense of glucose transport to the fetus or glucose usage by the placenta for cellular function and growth. Do you have this data available and could it be included if you do have this data? If not, could you please speculate in the discussion.

The reviewer raises an important and interesting point. Unfortunately, we did not quantify placental glucose transport to the fetus, placental glucose usage or fetal glucose levels. However, as recommended, we have speculated on their contribution in the revised discussion (lines 496-500).

2 While Slc2a1 was not impacted in the JZ, there might have been some indirect effects of changes in JZ glycogen content and endocrine function on labyrinth transport. Can you comment on how these impacts might have affected the labyrinth?

© 2022. Published by The Company of Biologists under the terms of the Creative Commons Attribution License (https://creativecommons.org/licenses/by/4.0/).

To address a potential indirect effect of Jz-∆ICR1 in the placental labyrinth, we assessed the expression of key glucose (*Slc2a1* and *Slc2a3*), as well as amino acid (system A; *Slc38a1, Slc38a2* and *Slc38a4*), transporters. As the labyrinth also performs an important role in regulating the fetal exposure of maternal glucocorticoids we also assessed the key glucocorticoid metabolising enzymes (*Hsd11b1* and *Hsd11b2*) in separated Lz tissues by qPCR (see Fig. 8). These data revealed a significant reduction in the expression of *Slc2a3* and *11bhsd1* by the Lz regardless of fetal sex, in response to Jz-∆ICR1 (Fig. 8B,C). These new results and their interpretation with regards to feto-placental phenotype with Jz-∆ICR1 has been included in the revised paper. Please see lines 308-318 in results and 512-533 in discussion.

As Jz expression of *Psg23* was highly induced in response to Jz-∆ICR1, we measured expression of the only known PSG receptor (*Cd9*) in the placental Lz to try and inform on the mechanisms by which indirect effects in the placenta may occur. However our data showed that while *Cd9* was expressed by the Lz, its expression was not significantly altered by Jz-∆ICR1, regardless of fetal sex (Figure S5, lines 318-322). Thus further work is required to understand the mechanisms by which Jz-∆ICR1 may indirectly affect the Lz.

3 One of your major findings was that psg23 expression was increased. It would have been great to see protein abundance of this gene but understand that there are unlikely to be any antibodies available to test this. You have alluded to the fact that changes to this gene may impact other aspects of placental function that have not been measured in this study but it is not clear why this gene was increased in the JZ while other peptides were not. Could you comment on this further in the discussion?

Ideally we would wish to quantify a wide array of placental proteins, including PSG23, but as the reviewer correctly states antibodies do not exist for the majority of these targets. Furthermore, such investigations would need to be undertaken in litters comprised entirely of either control or Jz-∆ICR1 to avoid the dilution of the Jz-∆ICR1 phenotype by the presence of control littermates. The explanation for why some hormone genes are altered by Jz-∆ICR1 and others are not is unknown. However, manipulations of other genes, like the PI3K pathway, through which IGF2 can signal also exerts changes in select placental hormone genes (Sferruzzi-Perri et al., 2016). There are also examples in the literature showing that manipulation of other imprinted genes, like *Phda2* (Tunster et al., 2015), *Peg3* (Tunster et al., 2018a), *Ascl2* (Tunster et al., 2018), and Cdk1nc (Tunster et al., 2011) have specific effects on placental hormone gene expression. We have added a comment about this in the revised discussion, please see lines 412-417.

- Sferruzzi-Perri AN, López-Tello J, Fowden AL, Constancia M. Maternal and fetal genomes interplay through phosphoinositol 3-kinase (PI3K)-p110α signaling to modify placental resource allocation. Proceedings of the National Academy of Sciences. 2016 Oct 4;113(40):11255-60.
- Tunster SJ, Creeth HD, John RM. The imprinted Phlda2 gene modulates a major endocrine compartment of the placenta to regulate placental demands for maternal resources. Developmental Biology. 2015 Oct 23;409(1):251-60.
- Tunster SJ, Boqué-Sastre R, McNamara GI, Hunter SM, Creeth HD, John RM. Peg3 deficiency results in sexually dimorphic losses and gains in the normal repertoire of placental hormones. Frontiers in cell and developmental biology. 2018a Sep 27;6:123.
- Tunster SJ, Van de Pette M, Creeth HD, Lefebvre L, John RM. Fetal growth restriction in a genetic model of sporadic Beckwith-Wiedemann syndrome. Disease models & mechanisms. 2018b Nov 1;11(11):dmm035832.
- Tunster SJ, Van de Pette M, John RM. Fetal overgrowth in the Cdkn1c mouse model of Beckwith-Wiedemann syndrome. Disease models & mechanisms. 2011 Nov;4(6):814-21.

4 You make a good point in the discussion about the potential impact of changes in the endocrine function of the placenta on maternal physiology. This in turn may impact the development of wild type littermates. Can you indicate if the placental weights and fetal weights of the wild type littermates in this model are similar to placental/fetal weights from this gestational age from previous studies?

© 2022. Published by The Company of Biologists under the terms of the Creative Commons Attribution License (https://creativecommons.org/licenses/by/4.0/).

We would be reluctant to compare fetal/placental weights across studies undertaken at different times. In order to make reliable comparisons all litters to be compared need to be generated contemporaneously. Our study was not designed to include such additional comparisons, which would dramatically increase animal usage to provide sufficient statistical power to assess the effect of genotype, sex and maternal environment (in addition to various levels of interaction).

5 You highlight that cells with the 100um2 size range are increased. Can you please be specific in the methods section as to how this range is assessed (ie. is this cells between 50 and 150?)

We thank the reviewer for pointing this out. We have added additional clarification of the size of cells (see line 219) and added the bin width size to the materials and methods (see lines 625-626)

6 How do the GC cells in the decidua differ from those in the JZ. Do these share similar lineage? Are they cells which have migrated and if so why would they not be impacted in this model? Can this be made clear in the manuscript.

The glycogen cell lineage emerges around D6.5 within the ectoplacental cone, undergoes rapid proliferation from D12.5 and from around D14.5 begin to migrate into the decidua. Both migratory and non-migratory glycogen cells share a common origin, although the mechanisms that regulate migration are currently unknown. Whilst we observed an increased volume of glycogen cells in the Jz, the total volume of glycogen cells in the decidua was not also increased. This may suggest that GC migration may be impacted by Jz-∆ICR1, which should be explored in future work. We have added additional clarification related to this in lines 383-389.

7 Why would the expression of cell markers not change if the stereology has demonstrated a change in the composition of the JZ? Would this be due to the fact that entire JZ increased in size and that each of the cell types likely contributed to this increase in JZ? It would appear that the stereology was performed on a smaller sample size using visual inspection which may be less accurate than using genetic markers that are known to correlate with abundance of a specific cell type. Could you comment further on the difference on outcomes based on gene expression compared to stereology.

The stereology and gene expression data measure subtly different aspects of the placental phenotype and thus cannot be directly compared. They each have their positives and limitations. The stereological assessment employed a robust and validated approach to quantifying gross volumes of the three placental regions (Db, Jz and Lz) and the various cell types that comprise the Db and Jz. However, even with this in mind, there may be subtle changes that are not detected or obscured by stronger effects. The qPCR analysis can also be used to indicate the abundance of cell types, using specific cell markers, but these genes can be responsive also to intrauterine cues, and so can also represent the expression of target genes at the cellular level. These factors may explain the incongruence between stereology and gene expression results. By assessing both, we obtain a larger picture of what effect our manipulation has on the endocrine profile of the placenta. Moreover, although the sample size for stereological analysis is slightly less than for gene expression, using a samples size of n=8/genotype/sex over 7 litters for molecular analysis is pretty good and within the normal sample size expected for this type of analysis and our experimental design.

Minor comments
Line 550- Please indicate the number of fetuses used per litter.

We have added further clarification between lines 611-615. A single placenta from each of the four possible sex and genotype combinations (male control; male Jz-∆ICR; female control; female Jz-∆ICR) from each litter was selected for further analysis, where possible. Where multiple placentas of the same sex and genotype combination were present in the same litter, the placenta closest to the litter mean for that combination was selected.

© 2022. Published by The Company of Biologists under the terms of the Creative Commons Attribution License (https://creativecommons.org/licenses/by/4.0/).

Second decision letter

MS ID#: DEVELOP/2021/199811

MS TITLE: Loss of imprinting of the Igf2-H19 ICR1 enhances placental endocrine capacity via sex-specific alterations in signalling pathways in the mouse.

AUTHORS: Bethany R.L. Aykroyd, Simon J Tunster, and Amanda N Sferruzzi-Perri
ARTICLE TYPE: Research Article

I am happy to tell you that your manuscript has been accepted for publication in Development, pending our standard ethics checks.

Reviewer 1

Advance summary and potential significance to field

The authors showed the importance of the ICR1 in the placental endocrine function.

Comments for the author

The authors addressed every one of the many concerns of the two reviewers. I am satisfied with this very nice revision.

Reviewer 2

Advance summary and potential significance to field

The authors have nicely addressed all of my comments.

Comments for the author

No further comments

© 2022. Published by The Company of Biologists under the terms of the Creative Commons Attribution License (https://creativecommons.org/licenses/by/4.0/).
